# An In Vivo Stable Isotope Labeling Method to Investigate Individual Matrix Protein Synthesis, Ribosomal Biogenesis, and Cellular Proliferation in Murine Articular Cartilage

**DOI:** 10.1093/function/zqac008

**Published:** 2022-02-25

**Authors:** Kamil A Kobak, Albert Batushansky, Agnieszka K Borowik, Erika Prado Barboza Lopes, Frederick F Peelor III, Elise L Donovan, Michael T Kinter, Benjamin F Miller, Timothy M Griffin

**Affiliations:** Aging and Metabolism Research Program, Oklahoma Medical Research Foundation, Oklahoma City, OK, 73104, USA; Institute of Heart Diseases, Wroclaw Medical University, Wroclaw 50-367, Poland; Aging and Metabolism Research Program, Oklahoma Medical Research Foundation, Oklahoma City, OK, 73104, USA; Aging and Metabolism Research Program, Oklahoma Medical Research Foundation, Oklahoma City, OK, 73104, USA; Aging and Metabolism Research Program, Oklahoma Medical Research Foundation, Oklahoma City, OK, 73104, USA; Aging and Metabolism Research Program, Oklahoma Medical Research Foundation, Oklahoma City, OK, 73104, USA; Aging and Metabolism Research Program, Oklahoma Medical Research Foundation, Oklahoma City, OK, 73104, USA; Aging and Metabolism Research Program, Oklahoma Medical Research Foundation, Oklahoma City, OK, 73104, USA; Aging and Metabolism Research Program, Oklahoma Medical Research Foundation, Oklahoma City, OK, 73104, USA; Oklahoma Center for Geroscience and Healthy Brain Aging, Department of Biochemistry and Molecular Biology, Department of Physiology, University of Oklahoma Health Sciences Center, Oklahoma City, OK, 73104, USA; Aging and Metabolism Research Program, Oklahoma Medical Research Foundation, Oklahoma City, OK, 73104, USA; Oklahoma Center for Geroscience and Healthy Brain Aging, Department of Biochemistry and Molecular Biology, Department of Physiology, University of Oklahoma Health Sciences Center, Oklahoma City, OK, 73104, USA; Research & Development, Veterans Affairs Medical Center, Oklahoma City, OK, 73104, USA

**Keywords:** extracellular matrix turnover, cellular turnover, cartilage protein synthesis, chondrocyte proliferation, deuterium labeling

## Abstract

Targeting chondrocyte dynamics is a strategy for slowing osteoarthritis progression during aging. We describe a stable-isotope method using in vivo deuterium oxide labeling and mass spectrometry to measure protein concentration, protein half-life, cell proliferation, and ribosomal biogenesis in a single sample of murine articular cartilage. We hypothesized that a 60-d labeling period would capture age-related declines in cartilage matrix protein content, protein synthesis rates, and cellular proliferation. Knee cartilage was harvested to the subchondral bone from 25- to 90-wk-old female C57BL/6J mice treated with deuterium oxide for 15, 30, 45, and 60 d. We measured protein concentration and half-lives using targeted high resolution accurate mass spectrometry and d2ome data processing software. Deuterium enrichment was quantified in isolated DNA and RNA to measure cell proliferation and ribosomal biogenesis, respectively. Most collagen isoforms were less abundant in aged animals, with negligible collagen synthesis at either age. In contrast, age altered the concentration and half-lives of many proteoglycans and other matrix proteins, including several with greater concentration and half-lives in older mice such as proteoglycan 4, clusterin, and fibronectin-1. Cellular proteins were less abundant in older animals, consistent with reduced cellularity. Nevertheless, deuterium was maximally incorporated into 60% of DNA and RNA by 15 d of labeling in both age groups, suggesting the presence of two large pools of either rapidly (<15 d) or slowly (>60 d) proliferating cells. Our findings indicate that age-associated changes in cartilage matrix protein content and synthesis occur without detectable changes in the relative number of proliferating cells.

## Graphical Abstract

**Graphical Abstract:**
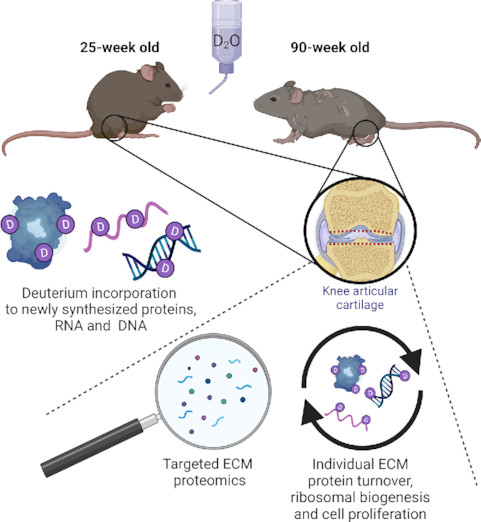
Assessment of fraction new over time reveals insight into proteostatic maintenance in muscle collagen.

## Introduction

Cartilage cellular stress and extracellular matrix degradation are central features of osteoarthritis (OA).^1^ During the course of OA, chondrocytes respond to joint stress in multiple ways that are associated with pathologic outcomes, including proliferation, senescence, cell death, and altered rates of extracellular matrix (ECM) synthesis and degradation.^2^ Ultimately, a net imbalance between ECM synthesis and degradation results in the loss of the mechanical and structural properties of articular cartilage. Understanding the relationship between chondrocyte dynamics and ECM turnover prior to and following the onset of OA may reveal how targeting chondrocyte dynamics (e.g., progenitor cell stimulation or senolytics) slows ECM degradation and OA progression.

Chondrocyte cellular turnover is altered during OA pathogenesis. Once skeletal growth ceases, the rate of chondrocyte proliferation significantly declines and most chondrocytes are considered post-mitotic,^3^ although some studies suggest that chondrocytes retain mitotic activity into adulthood.^1^,^4–6^ Following joint trauma and during the early stages of OA, chondrocyte proliferation increases in what has been interpreted as an attempted repair response.^7–10^ However, this transient increase in proliferation leads to a maladaptive spatial reorganization, loss of pericellular matrix of superficial chondrocytes,^11–13^ and the emergence of chondrocyte clones.^14^ OA is also associated with reduced chondrocyte proliferation and cell number through senescent and cell death pathways.^13,15^ With such a wide variety of cellular changes occurring over the course of disease, a broad range of therapies have been proposed, including methods to stimulate chondrocyte progenitor cells,^16^ eliminate senescent cells,^17^ and block apoptosis.^18^ A better understanding of the relationship between chondrocyte turnover and the synthesis of ECM proteins in vivo may provide important insight into strategies to promote the maintenance of articular cartilage.

Most studies have evaluated cartilage ECM protein turnover separately from chondrocyte proliferation. Early studies of ECM protein turnover used short-term radiolabeling to show that proteoglycan turnover in rabbit, canine and human cartilage was greater in the superficial zone compared to the deep zone and was similar between healthy and OA cartilage.^19^ In contrast, radiolabeling studies of cartilage collagen indicated an increase in the rate of collagen synthesis during the early stages of OA,^20–22^ consistent with an increase in C-propeptide levels of type II procollagen (CPII) observed during early OA.^23^ More recent insight into cartilage ECM turnover has come from evaluating the spontaneous clock-like accumulation of post-translational protein modifications, such as Asp racemization^24^ and Asn deamidation.^25,26^ These studies revealed OA-dependent, protein-specific, and joint-specific gradients in cartilage protein turnover without the use of exogenous labels.^24,25^ However, neither exogenous radiolabels nor endogenous molecular clock approaches have been applied to study the in vivo temporal and functional relationship between chondrocyte turnover and ECM protein synthesis rates.

The goal of this study was to establish a method to measure the rates of chondrocyte proliferation and absolute synthesis of individual cartilage proteins in the same tissue sample of mouse articular cartilage. The ability to perform these measurements simultaneously could inform the dependency between chondrocyte proliferation and protein turnover using only small amounts of tissue. To accomplish this goal, we used deuterium oxide (D_2_O) as a stable isotope labeling method. D_2_O is considered a universal tracer because the ubiquitous incorporation of water into most biochemical processes allows deuterium to accumulate in macromolecules through reactions of intermediary metabolism.^27^ Since D_2_O is functionally identical to normal water, labeling can be conducted through the drinking water for days, weeks, or even months, which is advantageous for slowly turning over tissues like cartilage. The fractional isotopic incorporation of deuterium into peptides, deoxyribose, and ribose reflects the synthesis of individual proteins, DNA and RNA, respectfully.^27^ DNA synthesis rate provides a direct measurement of cell proliferation over the labeling period.^27^ RNA synthesis rate measures ribosomal biogenesis, a key regulatory step for promoting anabolism. Finally, when D_2_O is used with targeted proteomics, it is possible to measure changes in concentration and synthesis rate of individual proteins.^27^ Therefore, from a single labeling experiment, multiple turnover processes can be measured simultaneously.

**Figure 1.**
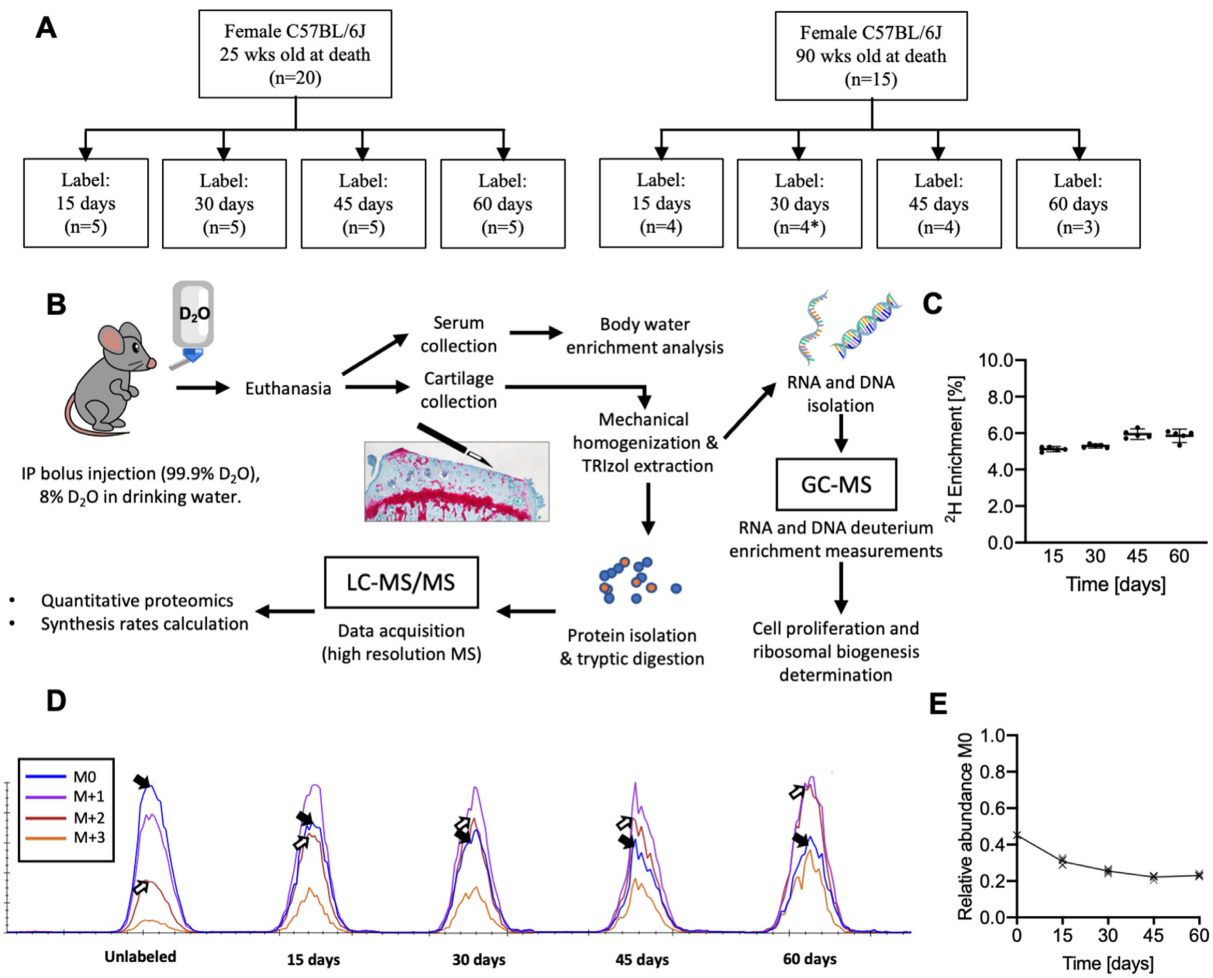
Experimental design and sample processing to investigate murine articular cartilage matrix protein synthesis, ribosomal biogenesis, and chondrocyte proliferation. (A) Overview of experimental design and D_2_O label duration sample sizes for each age cohort. *30-d labeling time point in 90-wk-old study group was excluded from analysis due to dosing concentration error. (B) D_2_O labelling and sample workflow description. (C) Body water enrichment in serum of 25-wk-old animals. Each point represents individual animal. Data are presented as mean ± 95%CI. (D) Representative isotopic distribution for one DCN peptide (ASYSAVSLYGNPVR) from an unlabeled sample and samples labeled with D_2_O for 15, 30, 45, 60 d in 25-wk-old animals. (E) Relative abundance of M0 in for the example DCN peptide (each x corresponds to an individual animal).

To evaluate this method in mouse articular cartilage, we treated female C57BL/6J mice with D_2_O for 15, 30, 45, and 60 d. We investigated two cohorts of mice that were 25 and 90 wks old at the completion of the labeling periods to provide an age-related perspective on changes in cellular proliferation, ribosomal biogenesis, individual protein content, and protein synthesis rates (Figure 1). We hypothesized that a 60-d labeling period would be sufficient to capture age-related declines in cartilage ECM protein content, protein synthesis, and cellular turnover.

## Materials and Methods

### Study Design and Animal Experimentation

All experiments were conducted in accordance with protocols approved by the AAALAC-accredited Institutional Animal Care and Use Committee at the Oklahoma Medical Research Foundation (OMRF). Female C57BL/6J mice (The Jackson Laboratory, Bar Harbor, ME, USA) were housed in the OMRF vivarium under the care of the Department of Comparative Medicine. Mice were group housed (≤5 animals/cage) in ventilated cages in a temperature-controlled room maintained at 22 ± 3°C on 14 h:10 h light/dark cycles with ad libitum access to chow and water. Mice were labeled as previously described.^28^ Briefly, mice were randomly assigned by cage to a deuterium oxide (D_2_O) labeling duration of 15, 30, 45, or 60 d (n = 3–5 per age and labeling duration). An overview of the study sample size and experimental design is provided in Figure 1(A and B). Three additional animals were euthanized prior to receiving D_2_O to determine the natural abundance of heavy isotopes in cartilage proteins. We initiated D_2_O labeling on different dates for each cage and age cohort so that animals were euthanized at either 25 or 90 wks of age. All mice received an initial i.p. bolus injection of 99% D_2_O followed by 8% D_2_O enriched drinking water for the designated labeling period. For tissue and blood collection, mice were anesthetized with isoflurane followed by exsanguination through cardiac puncture for euthanasia. Blood was collected and then centrifuged at 2000 g for 10 min. at room temperature. Serum was then aliquoted and frozen at −80°C until further analysis. Tibial and femoral articular cartilage from both hindlimbs were quickly harvested by gross dissection with a scalpel blade under a stereoscope and immediately frozen in liquid nitrogen. The extracted tissue reached to the superficial layer of subchondral bone (Figure 1B)

### Body Water Determination

To determine body water enrichment, 100 μL of serum was placed in the inner well of an o-ring cap of inverted screw-capped tubes and placed in a heat block for overnight distillation at 80°C. Distilled samples were diluted 1:300 in ddH_2_O and analyzed on a liquid water isotope analyzer (Los Gatos Research, Los Gatos, CA, USA) against a standard curve prepared with samples containing different concentrations of D_2_O.^29,30^ Body water enrichment occurred rapidly and reached a plateau of 5.5% by day 15 (Figure 1C).

### RNA/Protein/DNA Isolation

Tibial and femoral articular cartilage from the left and right limbs were pooled together and homogenized to a frozen powder using a TissueLyser (Qiagen). About 500 μL of TRIzol® Reagent (Thermo Fisher, Rockford, IL, USA) was then added to the powdered frozen cartilage. The homogenate was centrifuged at 16 000 g for 10 min at 4°C. The resulting supernatant was removed and 160 μL of chloroform was added. The mixture was then shaken vigorously for 30 s and centrifuged at 12 000 g for 15 min at 4°C. The solution separated into three phases: a transparent RNA-rich aqueous phase, a protein-rich TRIzol phase, and a DNA-rich interphase and pellet.

For RNA isolation, the upper aqueous layer was isolated, mixed with an equal amount of 100% ethanol, and left to incubate at room temperature (RT) for 10 min. The sample was transferred to a Zymo-Spin™ IC Column and purified in accordance with the manufacturer's protocol. RNA was eluted using 20 μL DNase/RNase-Free Water.

For protein isolation, 150 μL of 100% ethanol was added to the TRIzol interphase and mixed by inversion. The mixture was centrifuged at 2000 g for 10 min at 4°C. Protein supernatant was collected into a new tube with 400 μL of isopropanol, and the pellet was kept for DNA isolation. The protein supernatant was incubated for 10 min and centrifuged at 12 000 g for 10 min at 4°C to obtain a protein pellet, which was washed 3 times with 0.5 mL 0.3M guanidine-HCl in 95% ethanol for 20 min at RT and centrifuged at 7500 g for 5 min at 4°C. After the third wash, 1 mL of 100% ethanol was added, mixed, incubated at RT for 20 min, and centrifuged at 7500 g for 5 min. The supernatant was discarded, and the pellet was dried for 10 min at RT. To dissolve the protein pellet, 100 μL of 1% SDS was added and mixed by pipetting, vortex, and sonication on ice. An additional 100 μL of 1% SDS was added to the sample followed by incubation on a thermo-shaker at 50°C and 1200 rpm for 10 min. The sample was then centrifuged at 16 000 g for 10 min at RT, and the supernatant with solubilized protein was collected. Protein concentrations were measured using a detergent compatible reagent kit (BioRad DC Protein Assay), and the dissolved protein was prepared for mass spectrometry analysis.

For DNA isolation, the interphase and pellet were washed twice with 0.5 mL of 0.1 M sodium citrate in 10% ethanol, pH 8.5 and incubated for 30 min at RT. The mixture was then centrifuged at 2000 g for 5 min at 4°C. After discarding the supernatant, 0.75 mL of 75% ethanol was added, incubated for 15 min at RT, and centrifuged at 2000 g for 5 min at 4°C. The supernatant was discarded, and the DNA pellet was dried for 10 min and resuspended in 20 μL 8 mM NaOH.

### Quantitative Analysis of Targeted Protein Concentration and Isotope Incorporation

Protein samples were prepared as previously described.^31^ Briefly, 50 µg of total protein were taken for analysis. About 8 pmol BSA was added to the protein samples in 1% SDS as an internal standard. The total protein was desalted by precipitation in 1 mL of acetone overnight at −20°C. The protein pellet was solubilized in 50 µL Laemmli sample buffer and 20 µg protein was run in a 12.5% SDS-Page gel (BioRad Criterion system). The gels were fixed and stained with Coomassie blue (GelCode blue, Pierce Chemical Company). Each sample was cut from the gel as the entire lane and divided into smaller pieces. A standard in-gel digestion method was used.^32^ The gel pieces were washed to remove the Coomassie blue and then reduced in 10 mg/mL DTT,  alkylated in 35 mg/mL iodoacetamide,  and digested overnight with 1 µg trypsin per sample in 200 µL 10 mM ammonium bicarb. The mixture of peptides was extracted from the gel, evaporated to dryness in a SpeedVac, and reconstituted in 150 μL 1% acetic acid (v/v) for LC-tandem MS analysis.

Protein concentration and isotopic distribution were evaluated by LC-high resolution MS. We used a QEx Plus hybrid quadrupole-orbitrap mass spectrometry system (ThermoScientific), a splitless nanoflow HPLC system with autoinjector (ThermoScientific), and a 10 cm C18 column (Phenomenex Aeris 3.6 µm Peptide XB-C18 100A) packed in a fused silica electrospray tip (New Objective). About 5 µL sample volumes were injected and loaded onto the column at 1.5 µL/min with 0.1% formic acid. The column was eluted at 150 nL/min with a linear gradient of CH_3_CN in water with 0.1% formic acid (2% CH_3_CN to 65% CH_3_CN in 60 min). The orbitrap mass spectrometer acquired full scan mass spectra with a m/z resolution of 280 000. Ion source settings included a spray voltage of 1.5 kV, ion transfer tube temperature of 300°C, and positive ions mode.

A total of 36 ECM and cellular proteins were monitored in these experiments as listed in Table 1. The high resolution accurate mass (HRAM) analyses were managed through the program Skyline^33^ (Macoss laboratory) and included at least two unique peptides from each protein as verified from the peptide atlas database (http://www.peptideatlas.org/). Integrated chromatographic peak areas for each peptide were determined using the Skyline program. The response for each protein was calculated as the total integrated area for all peptides monitored for that protein. Data were analyzed as either the raw total integrated area or after normalization to the internal BSA standard to calculate protein concentration. Skyline was used to monitor and process data from each peptide.^33^ Targeted protein concentration was calculated as the geometric mean of all unique peptides, normalized to the BSA internal standard.

**Table 1.** Effect of age on articular cartilage protein concentration.

	Protein name	Fold-change (90 wk:25 wk)	Mean difference ($\times \ {10^{ - 2}}$)	95% CI of difference ($\times \ {10^{ - 2}}$)	*P*-value
*Proteoglycans*					
	ACAN	1.96	28.6	[17.4, 39.7]	0.0001^#^
	ASPN*	1.24	0.96	[-0.70, 2.62]	0.2389
	BGN*	0.76	-22.2	[-37.1, -7.29]	0.0057^#^
	DCN*	1.00	0.22	[-14.0, 14.5]	0.9746
	FMOD*	0.52	-54.4	[-68.1, -40.7]	<0.0001^#^
	FN1	1.89	13.6	[6.85, 20.43]	0.0009^#^
	LUM*	1.17	7.04	[-7.28, 6.65]	0.3086
	OGN*	0.79	-2.26	[-5.21, 0.70]	0.1285
	PRELP	0.64	-26.0	[-36.6, -15.3]	<0.0001^#^
	PRG4	2.02	7.59	[4.20, 11.0]	0.0004^#^
*Collagens*					
	COL1A1	0.85	-9.36	[-21.67, 2.95]	0.1261
	COL2A1	0.64	-24.8	[-34.7, -14.8]	<0.0001^#^
	COL6A1	0.98	1.16	[-16.2, 7.44]	0.8726
	COL6A2	1.01	0.56	[-11.23, 12.35]	0.9210
	COL6A3	0.85	-5.62	[-15.0, 3.73]	0.2226
	COL9A1	0.86	-0.66	[-1.31, -0.09]	0.0469
	COL11A1	0.76	-0.67	[-1.12, -0.23]	0.0053^#^
	COL12A1	0.73	-2.56	[-3.97, -1.14]	0.0011^#^
*ECM-related*					
	CHAD	0.76	-17.4	[-32.3, -2.6]	0.0229^#^
	CHIL3	0.38	-3.87	[-5.65, -2.09]	0.0001^#^
	CILP	0.57	-0.98	[-1.33, -0.64]	<0.0001^#^
	CLU	2.37	10.0	[7.00, 13.1]	<0.0001^#^
	COMP	0.62	-18.4	[-25.8, -11.1]	<0.0001^#^
	LMNA	0.91	-0.41	[-1.66, 0.83]	0.4943
	MATN1	0.56	-0.58	[-0.74, -0.42]	<0.0001^#^
	MATN3	0.60	-1.71	[-2.68, -0.75]	0.0011^#^
	MFGE8	0.43	-53.0	[-62.1, -43.9]	<0.0001^#^
	SPP1	0.59	-7.4	[-9.9, -4.9]	<0.0001^#^
	THBS1	0.72	-2.78	[-5.03, -0.53]	0.0186^#^
	VTN	1.11	1.68	[-2.75, 6.11]	0.4339
*Cellular proteins*					
	ACTBG	0.61	-20.0	[-31.5, -8.5]	0.0014^#^
	HSP90B	0.82	1.09	[-2.47, 0.3]	0.1163
	HSPA1A	0.57	-5.45	[-8.69, -2.21]	0.002^#^
	HSPA5	0.69	-2.72	[-4.61, 0.83]	0.0081^#^
	PPIA	0.54	-1.30	[-1.95, -0.64]	0.0004^#^
	TUBA	0.51	-8.37	[-12.2, -4.5]	0.0001^#^

Protein concentration was calculated as geometric mean of the two best-detected peptides, normalized to an internal BSA standard [pmol/mg total protein]. Mean difference was calculated as 90-wk minus 25-wk values. Comparison between groups was performed using paired *t*-test (two-tailed) with Welch's correction.

* Small leucine-rich proteoglycans (SLRPs).

# *P*<< 0.05i > following Benjamini–Hochberg FDR correction for multiple comparisons.

### Protein Synthesis Rate Calculations

Protein synthesis rates were determined using d2ome software,^32^ which allows for automated quantification of isotopomers of tryptic peptides (both endogenous mass and heavier deuterium-enriched species). Using d2ome, the protein synthesis rates were determined using the time-course of deuterium incorporation and a nonlinear regression fit model. d2ome makes these calculations by determining the rate of decline of the M0 isotopomer as the mass shifts from the M0 isotopomer to heavier mass isotopomers (e.g., M + 1, M + 2, M + 3, etc.) with incorporation of deuterium over time (Figure 1D and E). The individual protein synthesis rates were calculated using the mean value and pooled standard deviation of all peptides for a protein. To minimize the impact of variability within quantifying peptides, d2ome-implemented Grubbs’ outlier detection and removal was used. Protein half-lives were calculated from the equation τ^1/2^ = ln(2)/k, where k is an individual protein synthesis rate. Absolute protein synthesis was calculated by multiplying protein concentration by the protein synthesis rate.

An experimental error occurred with the concentration of D_2_O added to the drinking water of the 30-d labeling cohort for the 90-wk-old animals. Therefore, this labeling timepoint could not be used for analysis. To determine how this might affect our synthesis calculations, we conducted a d2ome analysis for 25-wk-old animals with and without the 30-d labeling timepoint. The differences were negligible (Supplementary Table 1); therefore, the 30-d labeling timepoint was excluded from the d2ome calculations in both age groups.

### Ribosomal Biogenesis and Cell Proliferation Determination

Ribosomal biogenesis and cell proliferation were determined by measuring the fractional incorporation of D_2_O in isolated RNA and DNA according to our previously published methods.^29,30^ Briefly, RNA was hydrolyzed, derivatized, and analyzed on an Agilent 7890A GC coupled to an Agilent 5975C MS. DNA was hydrolyzed, derivatized, and analyzed on an Agilent 8890 GC coupled to an Agilent 7010B TQ MS. Data were analyzed using MassHunter software. All analyses were corrected for natural isotope abundance with an unenriched pentafluorobenzyl triacetyl purine ribose/deoxyribose derivative standard.

### Statistical Analyses

Our study sample size of 3–5 animals per labeling duration cohort was based on prior studies using D_2_O labeling for measuring individual protein synthesis in other tissues. These studies utilized ≥ 4 labeling time points with n ≥ 3 independent biological samples per time point.^28,34,35^ For protein concentration measurements, all animals in a specific age group were considered as an individual biological replicate (n = 20 for 25-wk-old animals, n = 11 for 90-wk-old animals). All values were reported as mean ± 95% CI, and age-related differences in protein concentration, half-life, and absolute protein synthesis were evaluated by two-tailed Student's *t*-test with Welch's correction due to anticipated age-related changes in sample variances. In addition, age comparisons that retained significance following Benjamini–Hochberg FDR adjustment were noted as described in figure legends. GraphPad Prism version 8.3.0 (GraphPad Software, San Diego, California, USA) was used for calculations and data presentation.

## Results

### Effect of Age on the Concentration of Knee Articular Cartilage ECM Proteins

**Figure 2.**
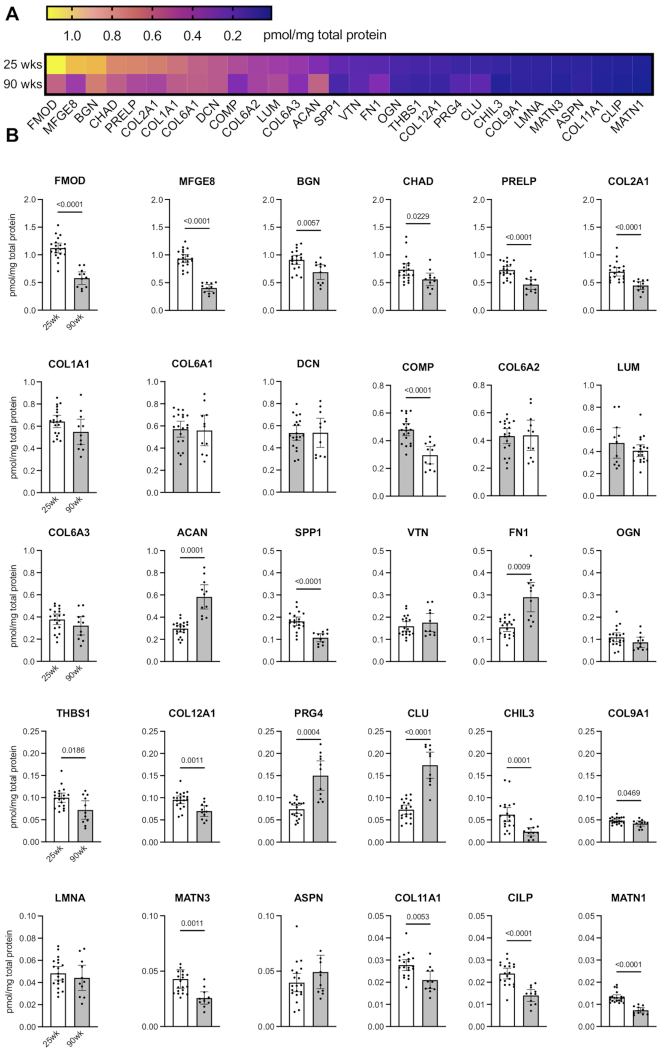
Age comparison of cartilage matrix protein concentration. (A) Protein concentration for all investigated extracellular matrix (ECM) proteins presented as a heat map. Proteins are ordered from the highest to lowest concentration in 25-wk old group. (B) Individual ECM protein concentration comparison between 25-wk (n = 20) and 90-wk (n = 11) old mice. Each point represents protein concentration for a single animal. Data are presented as mean ± 95%CI and expressed as pmol/mg total protein. Statistical comparison based on two-tailed Student's *t*-test with Welch's correction.

We developed a targeted ECM protein panel consisting of 30 proteins that were selected based on their established importance in cartilage composition and detectability from preliminary experiments. The panel included 10 proteoglycans, 8 collagen isoforms, and 12 other ECM related proteins (Figure 2, Table 1). We first evaluated the absolute concentration of these proteins and compared the difference in concentration between 25- and 90-wk-old animals. The concentration of targeted proteins spanned approximately three orders of magnitude, from a high range of 1.12 pmol/μg total protein for fibromodulin (FMOD) in 25-wk-old animals to the lowest detected concentration of 0.0073 pmol/μg total protein for matrilin 1 (MATN1) in 90-wk-old animals (Figure 2A, B). More than half of the proteins were less abundant in older animals, and a third of the proteins did not differ in concentration between the two ages (Figure 2; Table 1). Notably, four proteins were more abundant in the cartilage of aged animals (aggrecan [ACAN], fibronectin-1 [FN1], proteoglycan 4 [PRG4], and clusterin [CLU]).

Proteoglycan abundance varied markedly between 25- and 90-wks of age. For example, ACAN, FN1, and PRG4 concentration was approximately 2-fold greater in cartilage from older animals. The mean difference in protein concentration [95% CI of difference] was 28.6 [17.4, 39.7] * 10^–2^ pmol/μg total protein for ACAN, 13.6 [6.85, 20.4] * 10^–2^ pmol/μg total protein for FN1, and 7.59 [4.20, 11.0] * 10^–2^ pmol/μg total protein for PRG4 (Figure 2B, Table 2). In contrast, proline and arginine rich end leucine rich repeat protein (PRELP), which was the most abundant proteoglycan in cartilage from 25-wk-old mice, was 36% less abundant in 90-wk-old animals (-26.0 [-36.6, -15.3] * 10^–2^ pmol/μg total protein) (Figure 2B, Table 2). In addition, the highly abundant small leucine-rich proteoglycans (SLRPs) biglycan (BGN) and FMOD were approximately 25%–50% less abundant in cartilage from 90- versus 25-wk-old mice, whereas the less abundant SLRPs (i.e., asporin [ASPN], decorin [DCN], lumican [LUM], and osteoglycin [OGN]) were not different between the two ages (Figure 2B, Table 2).

**Table 2.** Effect of age on articular cartilage protein half-lives.

	Protein name	Number of peptides	Fold-change (90 wk:25 wk)	Mean difference ($\times \ {10^3}$)	95% CI of difference ($\times \ {10^3}$)	*P*-value
*Proteoglycans*						
	ACAN	4	22.0	22.1	[-89.9, 134.1]	0.6665
	ASPN*	2	0.54	-1.44	[-4.40, 1.52]	0.3245
	BGN*	6	1.52	0.88	[-1.97, 3.72]	0.5079
	DCN*	6	1.54	0.027	[0.012, 0.042]	0.0022^#^
	FMOD*	3	1.93	7.15	[-98.3, 112.6]	0.8820
	FN1	6	2.27	0.11	[0.050, 0177]	0.0026^#^
	LUM*	4	1.34	0.029	[0.003, 0.054]	0.0325
	OGN*	2	88.0	137	[-3942, 4216]	0.9411
	PRELP	4	1.70	0.27	[-0.033, 0.58]	0.0749
	PRG4	4	1.72	0.024	[0.018, 0.030]	<0.0001^#^
*Collagens*						
	COL1A1	2	45.0	135	[-1232, 1503]	0.8276
	COL2A1	4	–	–	–	–
	COL6A1	12	–	–	–	–
	COL6A2	7	2	121	[-2142, 2384]	0.9105
	COL6A3	15	1.57	6.00	[-40.2, 52.2]	0.7787
	COL9A1	4	–	–	–	–
	COL11A1	2	–	–	–	–
	COL12A1	4	–	–	–	–
*ECM-related*						
	CHAD	4	46.0	277	[-1152, 1695]	0.6766
	CHIL3	2	1.08	0.008	[-0.061, 0.078]	0.7965
	CILP	3	1.69	0.091	[-0.129, 0.310]	0.3906
	CLU	3	2.93	0.139	[0.077, 0.200]	0.0006^#^
	COMP	2	2.33	0.117	[0.046, 0.187]	0.0045^#^
	LMNA	7	1.12	0.008	[-0.012, 0.028]	0.3780
	MATN1	–	–	–	–	–
	MATN3	4	1.56	0.114	[-0.031, 0.259]	0.1153
	MFGE8	5	0.78	-0.055	[-0.490, 0.380]	0.7833
	SPP1	3	–	–	–	–
	THBS1	3	3.67	0.660	[-0.328, -1.65]	0.1650
	VTN	3	2.84	0.436	[0.030, 0.842]	0.0379

Protein half-lives were calculated from the decay constant for each protein obtained from d2ome software. Mean difference was calculated as 90-wk minus 25-wk values. Comparison between groups was performed using paired *t*-test (two-tailed) with Welch's correction.

* Small leucine-rich proteoglycans (SLRPs).

# *P*<<0.05i > following Benjamini–Hochberg FDR correction for multiple comparisons. Proteins that did not incorporate deuterium within the 60-d labeling duration are indicated by a dash.

For collagen (COL), the concentration of notable cartilage isoforms COL2A1, COL9A1, COL11A1 and COL12A1 were lower by 14–36% in 90- versus 25-wk-old animals (mean differences: -24.8 [-34.7, -14.8]; -0.66 [-1.33, -0.09]; -0.67 [-1.12, -0.23]; and -2.56 [-3.97, -1.14] * 10^–2^ pmol/μg total protein, respectively). In contrast, the concentration of COL1A1, COL6A1, COL6A2, and COL6A3 were similar between 25 and 90 wks of age (Figure 2B, Table 2). Finally, we observed a range of age-related effects on the concentration of 12 other ECM-related cartilage proteins. Most of these proteins were less abundant with age, including chondroadherin (CHAD), chitinase-like protein 3 (CHIL3), cartilage intermediate layer protein (CILP), cartilage oligomeric matrix protein (COMP), matrilin 1 (MATN1), matrilin 3 (MATN3), milk fat globule EGF and factor V/VIII domain containing protein (MFGE8), secreted phosphoprotein 1 (SPP1) and thrombospondin 1 (THSB1). Some of these proteins, such as COMP, are considered potential OA biomarkers due to their release into the serum following cartilage degradation. Notably, the concentration of CLU, which has previously been shown to be increased in OA cartilage, was 2.4-fold greater in the older cohort (10.0 [7.00, 13.1] * 10^–2^ pmol/μg total protein) as compared to younger mice.

### Aging Increases the Half-Life of Most Cartilage ECM Proteins

We calculated synthesis rates for all ECM proteins in our panel based on the rate of decline of the M0 isotopomers for each protein-specific peptide (Figure S1). The decline in M0 isotopomers due to D_2_O labeling varied considerably across our panel of proteins. For example, we observed obvious declines in M0 isotopomers during the 60-d labeling period in all peptides for some proteins, such as PRG4 and DCN; whereas other proteins showed variable peptide-specific declines in M0 isotopomers that diminished with age (e.g., ACAN, COL1A1, COL2A1) (Figure S1). Many collagen isoforms, however, showed trivial declines in M0 isotopomers of all peptides isolated from either 25 or 90-wk-old mice, indicating negligible synthesis of these proteins during the 60 d of labeling (e.g., COL6A1, Figure S1).

**Figure 3.**
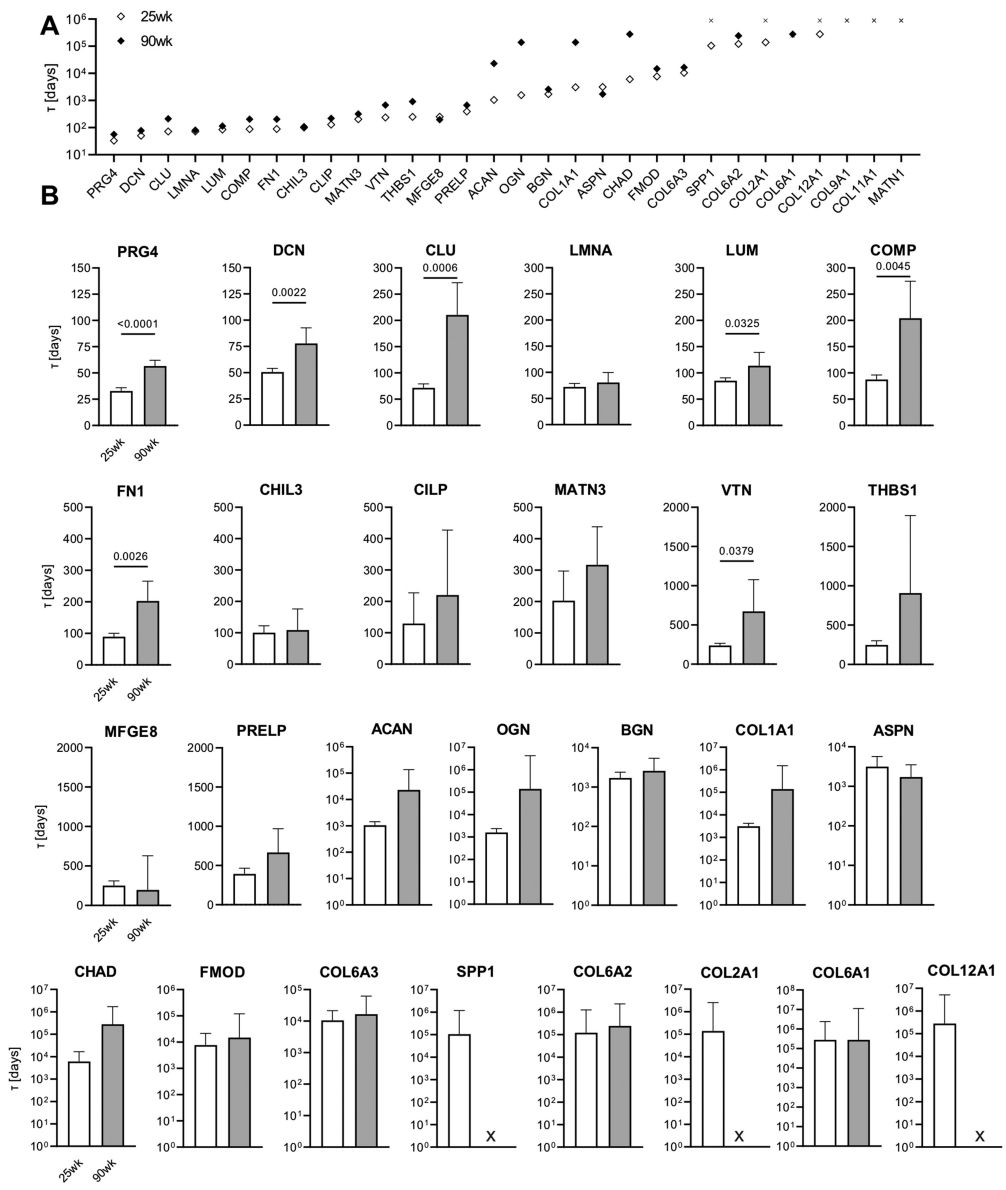
Age comparison of cartilage matrix protein half-lives. (A) Protein half-lives for all investigated ECM proteins, ordered from the shortest to the longest in 25-wk-old animals and presented in log scale. (B) Individual ECM protein half-life comparison between 25-wk (n = 20) and 90-wk (n = 11) old mice. Protein half-lives were calculated from the equation τ^1/2^ = ln(2)/k, where k is an individual protein synthesis rate. Data are presented as mean ± 95%CI [d]. “x” symbol indicates undetected deuterium incorporation within the 60-d labeling period. Statistical comparison based on two-tailed Student's *t*-test with Welch's correction.

To better understand the differences in age-related turnover of the investigated ECM proteins, we calculated the protein half-lives based on synthesis rates obtained for all proteins. Protein half-lives were highly heterogeneous, with the shortest half-life measured at 32.8 d for PRG4 in 25-wk-old animals to the longest half-lives being >760 yr for many of the collagen proteins (Figure 3A and B). Such extremely long half-life calculations result from negligible deuterium incorporation, which we observed for SPP1, COL2A1, COL6A1, COL12A1, COL9A1, COL11A1, and MATN1 in at least one age group (Figure 3A).

Protein half-lives were either unaltered or longer in duration in 90- versus 25-wk-old animals (Figure 3B, Table 2). Four out of 10 proteoglycans that we evaluated had longer half-lives with increasing age. For example, the half-lives of two SLRPs were longer by nearly 30 d in 90- versus 25-wk-old animals (mean difference in protein half-lives [95% CI of difference] was 27.3 [12.3, 42.3] d for DCN and 28.6 [2.9, 54.4] d for LUM). Similarly, the half-life of PRG4 was 23.7 [17.7, 29.8] d longer and the half-life of FN1 was 113.6 [50.3, 176.9] d longer in 90- versus 25-wk-old mice (Figure 3B, Table 2). Given the negligible decline in M0 isotopomers for nearly all collagen isoform peptides that we investigated, slight reductions in M0 isotopomers resulted in long half-life calculations, such as 3081 d (8.5 yr) for COL1A1 and 277 259 d (760 yr) for COL12A1 in 25-wk-old animals.

Most other ECM-related proteins had observable synthesis during the 60-d labeling period, with the half-lives generally longer in cartilage from the older animals (Figure S1). For example, the mean difference in half-lives for CLU, COMP and vitronectin (VTN) were longer in 90- versus 25-wk-old mouse cartilage by 138.5 [76.8, 200.3], 116.7 [46.0, 187.4] and 436.1 [30.4, 841.8] d, respectively (mean difference [95% CI]) (Figure 3B, Table 2).

### Absolute Cartilage ECM Protein Synthesis Declines with Age

**Figure 4.**
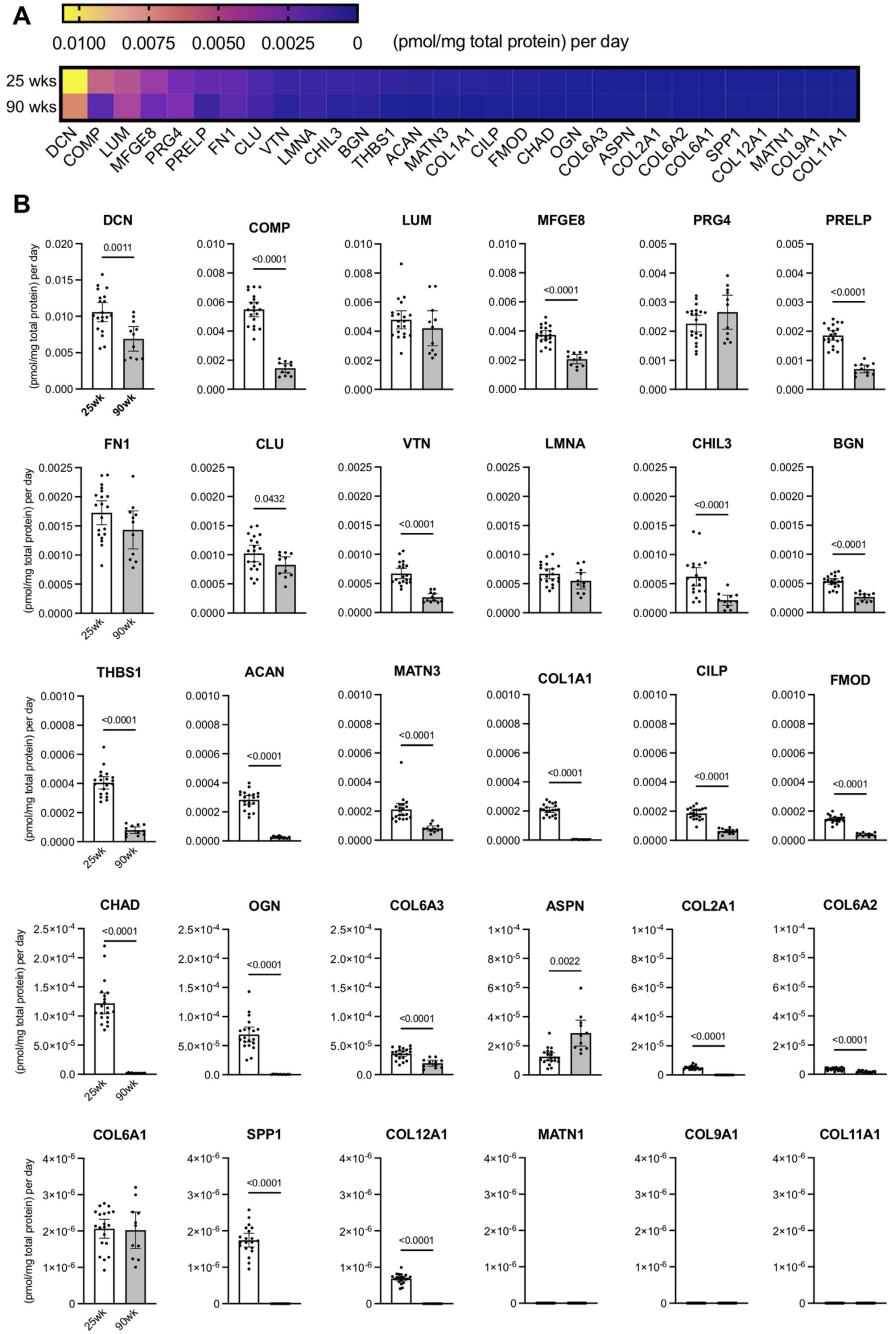
Age comparison of the absolute rate of targeted cartilage matrix protein synthesis. (A) Absolute rate of protein synthesis for all investigated ECM proteins presented as a heat map. Absolute protein synthesis rate was calculated by multiplying protein concentration by the protein synthesis rate. Proteins are ordered from the highest to lowest absolute protein synthesis rates from the 25-wk-old animals. (B) Individual absolute ECM protein synthesis rates from 25-wk (n = 20) to 90-wk (n = 11) old mice. Each symbol represents data for a single animal. Data bars are mean ± 95% CI [pmol/mg total protein per day]. Statistical comparison based on two-tailed Student's *t*-test with Welch's correction.

To obtain information about the absolute quantity of newly synthesized proteins (pmol/mg total protein per day), we calculated the absolute synthesis of each protein by dividing protein concentration by synthesis rate. Across the ECM proteins included in our targeted panel, we observed a wide range of absolute synthesis rates, from a high of 0.011 pmol/mg total protein per day for DCN to 6.89 * 10^–7^ pmol/mg total protein per day for COL12A1 (Figure 4A). Absolute protein synthesis could not be calculated for MATN1, COL9A1 and COL11A1 due to non-measurable synthesis rates in cartilage collected from both age groups.

Increasing age was associated with a smaller absolute amount of protein synthesized per day for most of investigated proteins (22 of 27) in 90-wk-old animals is synthesized in a smaller amount as compared to 25-wk group (Figure 4B, Table 3). Among SLPRs and proteoglycans with high levels of absolute daily protein synthesis at 25-wks of age, the levels were significantly lower with age such that the mean difference [95% CI of difference] between 90- and 25-wk old animals was -36.8 [-57.2, -9.85] * 10^–4^ pmol/mg total protein per day for DCN, -11.5 [-13.6, -9.46] * 10^–4^ pmol/mg total protein per day for PRELP, -2.67 [-3.35, -2.01] *10^–4^ pmol/mg total protein per day for BGN and -2.58 [-2.88, -2.27] * 10^–4^ pmol/mg total protein per day for ACAN. Collagen absolute synthesis levels were low compared to other ECM proteins, and they were also lower with age (e.g., COL1A1, COL2A1, COL6A2, COL6A3, and COL12A; Figure 4B, Table 3). Amounts of newly synthesized COMP and CLU were 74% and 19% lower with increasing age, respectively. Only the amount of newly synthesized ASPN was greater with age, with a mean difference of 0.16 [0.07, 0.25] *10^–4^ pmol/mg total protein per day in 90- versus 25-wk-old samples. The absolute daily synthesis of LUM, PRG4, FN1, lamin A (LMNA), and COL6A1 were similar in both age groups.

**Table 3.** Effect of age on the absolute synthesis rate of targeted articular cartilage matrix proteins.

	Protein name	Fold-change (90 wk:25 wk)	Mean difference ($\times \ {10^{ - 4}}$)	95% CI of difference ($\times \ {10^{ - 4}}$)	*P*-value
*Proteoglycans*					
	ACAN	0.09	-2.58	[-2.88, -2.27]	<0.0001^#^
	ASPN*	2.29	0.16	[0.07, 0.25]	0.0022^#^
	BGN*	0.50	-2.67	[-3.35, -2.01]	<0.0001^#^
	DCN*	0.65	-36.8	[-57.2, -9.85]	0.0011^#^
	FMOD*	0.27	-1.07	[-1.20, -0.94]	<0.0001^#^
	FN1	0.83	-2.94	[-6.65, 0.08]	0.1129
	LUM*	0.88	-5.85	[-19.0, 7.25]	0.3585
	OGN*	0.01	-0.69	[-0.81, -0.56]	<0.0001^#^
	PRELP	0.38	-11.5	[-13.6, -9.46]	<0.0001^#^
	PRG4	1.18	3.96	[-2.34, 10.3]	0.2006
*Collagens*					
	COL1A1	0.02	-2.05	[-2.23, -1.87]	<0.0001^#^
	COL2A1	0	-0.053	[-0.059, -0.045]	<0.0001^#^
	COL6A1	0.98	-0.00 042	[-0.0059, 0.0050]	0.8726
	COL6A2	0.51	-0.018	[-0.024, -0.012]	<0.0001^#^
	COL6A3	0.54	-0.165	[-0.23, -0.10]	<0.0001^#^
	COL9A1	–	–	–	–
	COL11A1	–	–	–	–
	COL12A1	0	-0.0069	[-0.0075, -0.0063]	<0.0001^#^
*ECM-related*					
	CHAD	0.17	-1.20	[-1.37, -0.02]	<0.0001^#^
	CHIL3	0.35	-4.01	[-5.78, -2.29]	<0.0001^#^
	CILP	0.35	-1.21	[-1.43, -1.00]	<0.0001^#^
	CLU	0.81	-1.96	[-3.86, -0.06]	0.0432
	COMP	0.26	-40.4	[-46.1, -34.7]	<0.0001^#^
	LMNA	0.82	-1.20	[-2.78, 0.38]	0.1279
	MATN1	–	–	–	–
	MATN3	0.38	-1.30	[-1.74, -0.21]	<0.0001^#^
	MFGE8	0.55	-16.7	[-20.7, -12.6]	<0.0001^#^
	SPP1	0	-0.017	[-0.019, -0.016]	<0.0001^#^
	THBS1	0.20	-3.25	[-3.72, -2.78]	<0.0001^#^
	VTN	0.39	-4.10	[-5.13, -3.06]	<0.0001^#^

Protein absolute synthesis was calculated by dividing protein concentration [pmol/mg total protein] by protein half-life [days]. Mean difference was calculated as 90 wk minus 25 wk values. Comparison between groups was performed using paired *t*-test (two-tailed) with Welch's correction.

* Small leucine-rich proteoglycans (SLRPs).

# *P*<<0.05i > following Benjamini-Hochberg FDR correction for multiple comparisons.

### D_2_O-based Estimates of Cellular Proliferation and Ribosomal Biogenesis are Consistent Across Age

**Figure 5.**
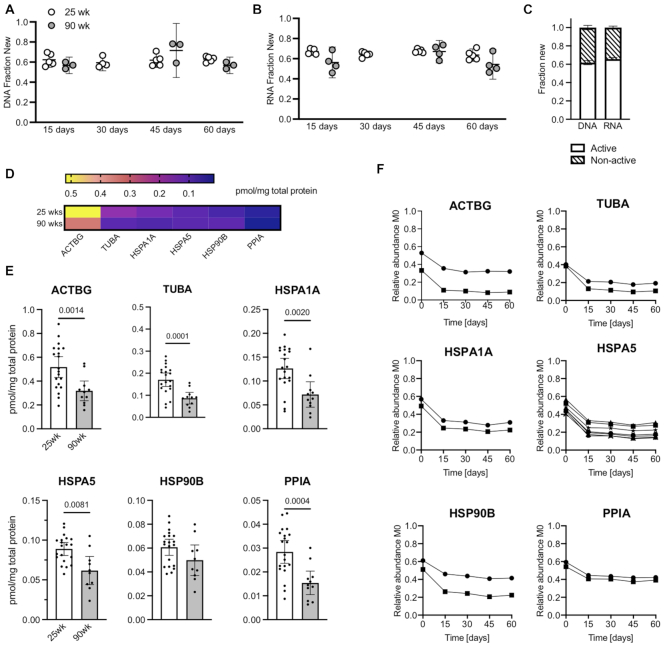
Age comparison of ribosomal biogenesis, cellular proliferation and intracellular protein deuterium incorporation. Fraction of newly synthesized (A) DNA and (B) RNA in extracted tissue samples based on deuterium incorporation during 15-, 30-, 45-, and 60-d labeling durations in 25- and 90-wk-old animals. DNA and RNA synthesis reflect rates of cell proliferation and ribosomal biogenesis, respectively. (C) Proportion of active and turnover-resistant pools of DNA and RNA in cartilage of 25-wk-old animals based on all deuterium labeling durations. Data are shown as mean ± 95% CI [Fraction New]. (D) Protein concentration of targeted intracellular proteins presented as a heat map. Proteins are ordered from the highest to lowest concentration from the 25-wk-old group. (E) Individual cellular protein concentration comparison between 25-wk (n = 20) and 90-wk (n = 11) old mice. Each symbol represents protein concentration for a single animal. Data are presented as mean ± 95%CI [pmol/mg total protein]. Statistical comparison based on two-tailed Student's *t*-test with Welch's correction. (F) Decline of relative abundance M0 for representative cellular proteins. Maximal deuterium incorporation occurred ≤ 15-d labeling duration.

We measured DNA and RNA synthesis in articular cartilage to obtain information about cell proliferation and ribosomal biogenesis, respectively. The newly synthesized fraction of DNA and RNA was similar across all labeling durations, indicating that D_2_O incorporation reached a plateau value in ≤ 15 d (Figure 5A and B). The fraction of newly incorporated DNA and RNA reached a plateau value at 0.6, which indicates that 60% of total DNA and RNA pools were turned over by 15 d and did not further increase with continued labeling (Figure 5C). Importantly, the plateau value was similar between the 25- and 90-wk-old animals, which indicates that chondrocyte proliferation and ribosomal biogenesis was similar at these two ages. To further evaluate this finding, we investigated the protein concentration and synthesis rates of common intra-cellular housekeeping proteins, such as actin (ACTBG), heat shock protein family A (Hsp70) member 1A and 5 (HSPA1A and HSPA5), heat shock protein 90 alpha family class B member 1 (HSP90B), peptidylprolyl isomerase A (PPIA) and tubulin alpha 1a (TUBA1A). First, we observed that 5 of these 6 cellular proteins were less abundant in the cartilage from 90- versus 25-wk-old animals (Figure 5D and E), suggesting an age-related decline in cartilage cellular content. In addition, the M0 isotopomer analysis of cellular protein peptides showed that deuterium incorporation plateaued by ≤ 15 d of labeling, consistent with DNA and RNA fractional incorporation of D_2_O (Figure 5F). The implication of this finding is that intra-cellular protein synthesis is largely coupled to cellular replication.

## Discussion

This report is the first to describe a method for simultaneously measuring individual protein synthesis, ribosomal biogenesis, and cellular proliferation in one sample of murine knee articular cartilage. By integrating in vivo D_2_O labeling with GC-MS and high-resolution LC-MS/MS analyses, we show that it is possible to measure the concentration and synthesis rates of targeted cellular and extracellular cartilage proteins as well as cell proliferation and ribosomal biogenesis. Although there was minimal synthesis of most collagen isoforms during the 60-d labeling period at 25- and 90-wks of age, many proteoglycans and other ECM-related proteins were newly synthesized over this labeling period. We hypothesized that 60 d of labeling would be sufficient to capture the age-related declines in cartilage ECM protein synthesis and chondrocyte turnover. Indeed, we observed a decline in the absolute synthesis of many cartilage ECM proteins between 25- and 90-wks of age. However, we were not able to compare age-related changes in ribosomal biogenesis or cellular proliferation because RNA and DNA deuterium incorporation rates plateaued at 60% by our shortest labeling duration (15 d) in both age groups. Nevertheless, these data were surprising because they indicate that approximately 60% of murine knee chondrocytes replicate over a 15-d period. Thus, our findings support the use of in vivo D_2_O labeling to investigate the rate of synthesis of targeted cartilage proteins and compare these rates to the dynamics of ribosomal biogenesis and cellular proliferation.

The findings of this in vivo D_2_O labeling study support the conclusions of prior studies of cartilage protein turnover and offer new insight into cartilage ECM proteostasis. As with prior radiocarbon dating,^36^ protein deamidation molecular clock,^25^ and SILAC labeling^37^ studies, D_2_O labeling showed that proteoglycan proteins have substantially shorter half-lives compared to collagen proteins and that several collagen isoforms show no appreciable turnover. Each of these methods for evaluating protein turnover offers different advantages in terms of sensitivity, specificity, cost, and safety. For example, molecular clock-based approaches may take advantage of different types of endogenous post-translational modifications with different clock rates to evaluate turnover in both short- and long-lived proteins without the need for exogenous labels. However, identifying peptides that accumulate modifications at a consistent clock-like rate requires knowledge of the protein crystal structure or *in silico* structural models, which may limit both the proteins and peptide-specific protein regions available for analysis.

Conversely, isotopic labelling, such as with SILAC and D_2_O, allows for direct measurements of protein synthesis by evaluating a broad range of protein-specific peptides. Furthermore, the use of exogenous labels allows turnover to be measured during defined periods of time. There are several distinctions between SILAC and D_2_O labeling methods. For example, SILAC labeling uses a pulse-chase method versus our continuous D_2_O labeling approach. The loss of heavy label following the SILAC pulse period involves both the incorporation of unlabeled amino acids and the breakdown of labeled amino acids to measure protein turnover. A disadvantage of a single time point labeling method is that it does not determine if or when a labeling plateau occurs. As we previously reported,^38^ protein turnover calculations are affected by dynamic protein pool size, and ECM protein label incorporation often plateaus at far less than 100%. In contrast, the D_2_O labeling method used in this study provided continuous labeling during the indicated time period and included multiple time points to determine the labeling plateau. Despite these methodological differences, our current study and a recent SILAC-based study^37^ both showed greater synthesis rates in proteoglycans compared to collagens and slower protein synthesis rates in cartilage from older mice. Both methods showed reduced synthesis rates of several proteins, including CLU, PRG4, COMP, and SPP1, in cartilage from older animals.

Of the proteoglycans included in our analysis, PRG4 had the shortest half-life at 33 d in 25-wk-old animals. PRG4 is primary produced by superficial zone chondrocytes. It is secreted into the synovial fluid and facilitates joint lubrication and energy dissipation under load. PRG4 is also expressed by chondrocyte progenitor cells,^39^ and increased PRG4 is chondroprotective.^40^ *Prg4* expression in murine knee joint tissues is reduced 50% between 10 and 95-wks of age,^41^ suggesting that an age-related reduction in PRG4 may contribute to an increase in OA risk. However, we found that the absolute synthesis rate of PRG4 in cartilage did not change between 25- and 90-wk-old animals. Moreover, the half-life nearly doubled with increasing age, and cartilage PRG4 content was 2-fold greater in the older animals. These findings suggest that there are age-related changes in pathways regulating PRG4 proteostasis, which may have important biological implications for age-related changes in synovial fluid properties and joint inflammation.^42^

Impaired protein homeostasis (proteostasis) is a hallmark of aging and is thought to contribute to the age-related increase in OA risk.^43^ For example, a decline in cartilage ECM protein synthesis is considered one reason that cartilage structure and mechanical properties diminish with age.^44^ It is important to note, however, that an imbalance in protein synthesis and degradation may manifest as either a net decline or increase in protein concentration. In the current study we found that despite a reduced or unchanged rate of synthesis of some proteins with age (e.g., ACAN, FN1, PRG4, and CLU), their concentration was greater in the cartilage of older mice. There are several proteostasis-related explanations for this outcome. One possibility is a reduced expression or activity of proteolytic enzymes that degrade these proteins. An additional possibility is that a fraction of these proteins may became resistant to turnover. Resistance to proteolysis is a known feature of aging.^38^ We recently showed that skeletal muscle collagen becomes resistant to turnover with age.^38^ Although in the present study the concentration of most of investigated collagen isoforms declined with age, collagens and other investigated proteins may become resistant to proteolysis with age due to the accumulation of post-translational modifications and/or cross-linking.^45^ Previous clock-based analyses of ACAN turnover identified short-lived (G3) and long-lived (G1) ACAN fragments in articular cartilage related to the retention of the N-terminus (G1) region in the cartilage matrix following metalloproteinase or ADAMTS proteolysis.^24,25^ Although we did not set out to examine peptide-specific differences in protein turnover, the ACAN peptides included in our analysis were in the G1 and G2 regions, and the half-life measurement was substantially greater for G1 versus G2. Therefore, additional insight may be gained into ECM proteostasis in future studies by intentionally selecting peptides to evaluate variation in region-specific protein turnover.

We made two assumptions that potentially impact the calculated rates of synthesis. First, we calculated protein half-lives with the assumption that the proteins were in a steady state, which means that we assumed that protein mass did not change during the period of labeling. During steady state, synthesis equals breakdown, and half-life can be calculated from the rate of synthesis. Although the concentration of some proteins changed between 25 and 90 wks of age, the amount of change estimated to have occurred during the longest labeling timepoint (60 d) is within our measurement error if we assume a linear change in protein mass from 25 to 90 wks (455 d). Therefore, we feel that this assumption is reasonable. The second assumption is that the size of the dynamic pool for each protein (or reciprocally the size of the protein pool resistant to turnover)^38^ is not different between mice 25 and 90 wks of age. We have demonstrated previously that the dynamic protein pool of skeletal muscle collagen becomes smaller with age.^38^ Further, if this decrease in dynamic pool size is not accounted for when comparing two conditions, the results can be appear to be opposite of the true outcome.^38^ In the present study, this concern does not appear to be an issue for the collagens since all the collagens had negligible turnover. However, it is possible that some of the non-collagen ECM proteins could become resistant to turnover with age, thus impacting their calculated rates. To guard against this potential error, we inferred plateau values from the M0 decay curve, which for most proteins appear to be at a similar value for both 25- and 90-wk-old mice. As future studies expand the scope of proteins evaluated in cartilage, potential changes in protein pool size should be rigorously evaluated.

Although we did not evaluate OA-related outcomes by histology, many of the age-related changes in cartilage protein concentrations may be related to OA pathology. We observed lower concentrations of numerous cartilage matrix proteins in older animals, including FMOD, BGN, CHAD, PRELP, COL2A1, COMP, COL9A1, MATN1, MATN3, COL11A1, CILP, and CHIL3. Many of these proteins are cleaved by proteases that are upregulated in cartilage during OA progression.^46^ For example, COMP is elevated in serum and synovial fluid with OA,^47^ and our findings are consistent with a recent proteomic analysis of human cartilage showing a reduction in cartilage COMP with OA.^25^ We also observed lower concentrations of the protein MFGE8 in the cartilage of older mice. MFGE8 is decreased in OA cartilage, and intra-articular injection of a neutralizing MFGE8 antibody following meniscal destabilization increased cartilage erosion, biomarkers of chondrocyte senescence, and synovial hyperplasia in mice, suggesting an OA-protective role for MFGE8^48^. Finally, the lower concentration of cellular proteins in 90- versus 25-wk-old mouse cartilage is consistent with a loss of cellularity.^44^ Accounting for age-related changes in cellularity when evaluating protein synthesis calculations suggests the declines in cartilage ECM protein synthesis may be attributed as much to a loss of cellularity as to a decline in cellular anabolic function with increasing age. Although a limitation of our study is the inability to distinguish between aging and OA-related changes that contribute to these findings, a validation experiment of samples from 6, 12, 18, and 24-mo-old female C57BL/6J mice showed that several of these age-related changes occurred prior to 18-mo of age (Supplemental Materials, Figure S2).

One important advantage of D_2_O labeling is the ability to evaluate cell proliferation and ribosomal biogenesis. Chondrocytes are generally considered to be post-mitotic.^3^ However, our DNA and RNA D_2_O incorporation data suggest that only about 40% of cells in our cartilage extracts do not proliferate over a 60-d period. The similar levels of RNA and DNA D_2_O incorporation suggest that most of newly synthesized ribosomes are due to cell proliferation. In contrast, approximately 60% of the cells in our cartilage samples undergo proliferation within a 15-d period. These findings were similar in both 25- and 90-wk-old mice, and they are substantially different from the only prior study using D_2_O labeling to evaluate chondrocyte proliferation in vivo.^49^ In that study, Li and colleagues reported that 44% of chondrocytes harvested from the medial tibial plateau of male Sprague Dawley rats had proliferated after 1 yr of labeling. Furthermore, the short-term labeling timepoints indicated that only 10% of chondrocytes proliferated within 15 d of labeling.^49^ There are several potential reasons why our analysis of cellular proliferation in mice was so different from rats. One possibility is that our cartilage tissue extracts included a population of more proliferative cell types present at the cartilage-bone interface as our method of tissue collection made it difficult to exclude the superficial layer of subchondral bone. Another possibility is the difference in methodology used to isolated DNA.^49^ For example, the method used by Li and colleagues—tissue sample fractionation and multi-step enzymatic digestion on cellulose column—may result in the loss of DNA during serial column elutions.^49^ It is also possible that differences in sex, species, and tissue harvest locations influenced these results. Our DNA and RNA-based evidence for rapid cellular turnover is further supported by our analysis of intra-cellular proteins, which showed that maximal D_2_O incorporation was reached within 15 d for all six cellular proteins (ACTBG, TUBA, HSPA1A, HSPA5, HSP90B, and PPIA).

Previous studies support our observation of distinct pools of rapidly and slowly proliferating chondrocytes. For example, Hunziker and colleagues showed that chondrocyte proliferation was associated with articular cartilage zonal distribution in developing rabbit cartilage.^50^ They used bromodeoxyuridine (BrdU) staining to identify slowly proliferating cells in the superficial zone and ^3^H-thymidine autoradiography to identify rapidly proliferating cells in the transitional and upper radial zones.^50^ ^3^H-thymidine labeling was also used in rabbits to show that chondrocyte proliferation is significantly increased following cartilage trauma.^6^ Although the small volume of murine knee cartilage tissue precludes that ability to evaluate zone-based differences in chondrocyte proliferation using the current method, D_2_O labeling could be used to evaluate acute changes in proliferation following joint trauma or treatment. Since the fraction new of DNA was already plateaued at 15 d, future studies will need shorter labeling durations (e.g., 1, 5, and 7 d) to capture differences in synthesis rates between treatment groups.

## Study Limitations

There are several important limitations to our study. First, we only conducted our study in female mice. Sex differences in the pathophysiology of OA suggest that our results will not completely replicate in male mice. However, the main goal of this study was to develop methodology to investigate differences in the ECM protein and cellular turnover in the same sample of murine articular cartilage. Second, we acknowledge that the age-based comparisons in this study may also include changes due to OA pathology. Unfortunately, it was not possible to collect the required tissue for the proteomic analysis and perform a histological analysis on the same joint samples. Third, we prioritized proteins for targeted analysis based on detectability and prior evidence showing involvement in cartilage function and mechanical properties. A discovery proteomic analysis combined with D_2_O labeling would likely identify more proteins with age-related changes in protein synthesis, although we would have been limited to relative rather than quantitative protein calculations. Fourth, a labeling period of 60 d was too short to detect the synthesis of several collagen isoforms. Although a longer labeling duration might address this limitation, the synthesis rates appear slow enough that the biological significance of measuring these very long half-lives may be negligible. Fifth, an increase in collagen crosslinking with age could decrease the solubility of total collagen and contribute to the observed loss of collagen content with age. Sixth, we failed to capture potential differences in chondrocyte proliferation and ribosomal biogenesis rates between the two age groups because of the surprisingly fast turnover that reached a plateau by the first timepoint. Thus, shorter labeling durations (<15-d) are needed to compare age-related differences in these rates. Finally, we performed our measurements in full-thickness articular cartilage harvested from both knees. This approach likely included portions of the superficial layer of subchondral bone. The whole-tissue approach also prevented that ability to examine zone-dependent differences in protein synthesis or cellular turnover.

## Conclusions

This study describes an in vivo D_2_O labeling method to simultaneously investigate the dynamics of ECM and cellular turnover in murine knee articular cartilage. We found that a 60-d labeling period was sufficient to detect a decline in the synthesis of many cartilage ECM proteins between 25- and 90-wks of age. Measurements of the relative proportion of proliferating cells were not different between 25 and 90-wks of age, suggesting that the age-associated changes in matrix protein content and synthesis occur independent of changes in cellular proliferation. A better understanding of the relationship between chondrocyte turnover and the synthesis of ECM proteins in vivo may provide important insight for evaluating strategies to promote the maintenance of articular cartilage, such as stimulating chondrocyte progenitor cells, eliminating senescent cells, or blocking apoptosis.

## Role of the Funding Source

Supported by the NIH (R01AG049058, P30GM114731, P20GM139763, R56AG067754, and P30AG050911) and the Department of Veterans Affairs (I01BX004666, 1I01BX004882). The content is solely the responsibility of the authors and does not necessarily represent the official views of the National Institutes of Health or the Department of Veterans Affairs. The funding sources played no role in the conduct, writing, or submission of the manuscript for publication.

## Supplementary Material

zqac008_Supplemental_File

## Data Availability

Peptide sequences and synthesis rates are included in this published article (and its supplementary information files). Kinetic proteomic data are available from the Zenodo.org database (10.5281/zenodo.5911976).
