# Supplementary material for: An In Vivo Stable Isotope Labeling Method to Investigate Individual Matrix Protein Synthesis, Ribosomal Biogenesis, and Cellular Proliferation in Murine Articular Cartilage
Source: Function (Oxf). 2022 Feb 25;3(2):zqac008. doi: 10.1093/function/zqac008 (PMC8991031; doi:10.1093/function/zqac008)
Supplement: zqac008_Supplemental_File [file zqac008_Supplemental_File.docx]

**Supplementary material**

**An *in vivo* stable isotope labeling method to investigate individual matrix protein synthesis, ribosomal biogenesis, and cellular proliferation in murine articular cartilage**

**Figure S1.** Time-dependent M0 relative abundance graphs of peptides specific for DCN, ACAN, COMP, CLU, COL2A1, COL6A1, ACTBG, HSPA1A in 25- and 90-week-old animals. Relative abundance of M0 is showed for unlabeled animals and after 15, 30, 45 and 60 days of D_2_O labeling. Note, 30-day labeling data for 90-week-old animals was excluded due to a technical error. Each symbol represents a different protein-specific peptide.

**DCN**

**ACAN**

**COL2A1**

**COL6A1**

**CLU**

**COMP**

**ACTBG**

**HSPA1A**

**B**

**A**

**D**

**C**

**F**

**E**

**H**

**G**

25wk

90wk

25wk

90wk

25wk

90wk

25wk

90wk

25wk

90wk

25wk

90wk

25wk

90wk

25wk

90wk

**Figure S2.** Protein abundance of targeted extracellular matrix (ECM) proteins in knee articular cartilage harvested from 6, 12, 18, and 24-month-old female C57BL/6 mice (n=8-9 per age). Protein abundance was based on ≥2 protein-specific peptides analyzed as described in the Material and Methods, with the exception that values were normalized to the total ion current. Data shown as mean ± 95% CI. Age-related changes in abundance were evaluated by Kruskal-Wallis statistical test.

**Table S1.** Synthesis rate of each protein-specific peptide for 25- and 90-week-old animals.

|  |  |  | **25-week-old** | | **90-week-old** | |
| --- | --- | --- | --- | --- | --- | --- |
| **Protein** | **Peptide sequence** | **Charge** | **Mean** | **SD** | **Mean** | **SD** |
| BGN | LGLGHNQIR | 2 | 0 | 0 | 0 | 0 |
| BGN | LGLGHNQIR | 3 | 0 | 0.0000025 | 0 | 0 |
| BGN | AFSPLR | 2 | 0 | 0 | 0 | 0 |
| BGN | GVFSGLR | 2 | 0 | 0 | 0 | 0 |
| BGN | VPAGLPDLK | 2 | 0 | 0 | 0 | 0 |
| BGN | IQAIELEDLLR | 2 | 0.00244 | 0.0007275 | 0.00161 | 0.0009975 |
| CHAD | EVAAGAFR | 2 | 0 | 0 | 0 | 0 |
| CHAD | LLNLQR | 2 | 0 | 0 | 0 | 0 |
| CHAD | NQLSSYPSAALSK | 2 | 0 | 0.00018 | 0 | 0 |
| CHAD | SIPDNAFQSFGR | 2 | 0.00003 | 0.000205 | 0.00001 | 0.0000025 |
| CHAD | FSDAAFSGVTTLK | 2 | 0.00035 | 0.00052 | 0 | 0.0000325 |
| CHAD | NNFPVLAANSFR | 2 | 0.00008 | 0.000425 | 0 | 0.000015 |
| CHIL3 | TGIGAPTISTGPPGK | 2 | 0.0095 | 0.002455 | 0.00974 | 0.0028325 |
| CHIL3 | DYEALNGLK | 2 | 0.00432 | 0.0030275 | 0.00302 | 0.0072975 |
| CHIL3 | HLFSVLVK | 2 | 0 | 0.0005575 | 0 | 0.0011375 |
| CILP | IVGPLEVNVR | 2 | 0.00213 | 0.0007275 | 0 | 0.000005 |
| CILP | FNPNAIGVPQPYLNK | 2 | 0.00916 | 0.0012525 | 0.00488 | 0.0025625 |
| CILP | LVLTFVDR | 2 | 0.00476 | 0.01256 | 0.00456 | 0.0066875 |
| COL2A1 | TVIEYR | 2 | 0 | 0 | 0 | 0 |
| COL2A1 | FTYTALK | 2 | 0 | 0 | 0 | 0 |
| COL2A1 | SLNNQIESIR | 2 | 0 | 0.0000025 | 0 | 0 |
| COL2A1 | NSIAYLDEAAGNLK | 2 | 0.00002 | 0.00031 | 0 | 0.000245 |
| COL6A2 | NLNEQGLR | 2 | 0 | 0 | 0 | 0 |
| COL6A2 | DIANSPHELYR | 2 | 0.00001 | 0.0001825 | 0.00001 | 0.0000375 |
| COL6A2 | DIANSPHELYR | 3 | 0 | 0.0000325 | 0 | 0.0000025 |
| COL6A2 | VNSLSSFK | 2 | 0 | 0 | 0 | 0 |
| COL6A2 | LFAVAPNR | 2 | 0 | 0 | 0 | 0 |
| COL6A2 | FAYNQLIK | 2 | 0 | 0 | 0 | 0 |
| COL6A2 | VGVVQYSHEGTFEAIR | 3 | 0.00003 | 0.000175 | 0.00001 | 0.0000825 |
| COL6A3 | NILTSSTGSR | 2 | 0 | 0 | 0 | 0 |
| COL6A3 | ELPNIEER | 2 | 0 | 0 | 0 | 0 |
| COL6A3 | IASNSATAFR | 2 | 0 | 0 | 0 | 0 |
| COL6A3 | DQNVFVSQK | 2 | 0 | 0 | 0 | 0 |
| COL6A3 | SSDAVAGPASSLK | 2 | 0 | 0 | 0 | 0 |
| COL6A3 | VFAVGVR | 2 | 0 | 0 | 0 | 0 |
| COL6A3 | VALVQYSDR | 2 | 0 | 0 | 0 | 0 |
| COL6A3 | VVESLDVGPDR | 2 | 0 | 0 | 0 | 0 |
| COL6A3 | ALEFVAR | 2 | 0 | 0 | 0 | 0 |
| COL6A3 | NAGPEFQYIR | 2 | 0 | 0 | 0 | 0 |
| COL6A3 | VAIAQFSDDVR | 2 | 0 | 0.000005 | 0 | 0.0000125 |
| COL6A3 | ELGTIQQVISER | 2 | 0.00001 | 0.000015 | 0.00001 | 0.000345 |
| COL6A3 | VPQIAFVITGGK | 2 | 0 | 0.00004 | 0 | 0.0000025 |
| COL6A3 | ISLSPEYVYSVSTFR | 2 | 0.00001 | 0.000005 | 0.00001 | 0.0001425 |
| COL6A3 | IGDLQSQIVSLLK | 2 | 0.00097 | 0.0004825 | 0.00061 | 0.000505 |
| COMP | AVAEPGIQLK | 2 | 0.00436 | 0.000935 | 0.0011 | 0.0009625 |
| COMP | ELQETNAALQDVR | 2 | 0.01149 | 0.0017675 | 0.00569 | 0.0021075 |
| COMP | EITFLK | 2 | 0.00001 | 0.0000375 | 0 | 0 |
| FN1 | GDSPASSKPVSINYK | 2 | 0.01142 | 0.00232 | 0.00558 | 0.00207 |
| FN1 | GDSPASSKPVSINYK | 3 | 0.00734 | 0.00213 | 0.00245 | 0.0014825 |
| FN1 | STTPDITGYR | 2 | 0.00249 | 0.0010575 | 0.00009 | 0.000425 |
| FN1 | ITGYIIK | 2 | 0 | 0 | 0 | 0 |
| FN1 | APITGYIIR | 2 | 0.00034 | 0.000825 | 0 | 0.0000025 |
| FN1 | ATGVFTTLQPLR | 2 | 0.00899 | 0.0015875 | 0.00512 | 0.0014825 |
| FN1 | NLQPGSEYTVTLVAVK | 2 | 0.01606 | 0.002065 | 0.00727 | 0.0020725 |
| LMNA | ITESEEVVSR | 2 | 0.00543 | 0.00122 | 0.00543 | 0.00281 |
| LMNA | SGAQASSTPLSPTR | 2 | 0.01195 | 0.0008925 | 0.01108 | 0.0032 |
| LMNA | AAYEAELGDAR | 2 | 0.00748 | 0.00138 | 0.00663 | 0.0021625 |
| LMNA | SLETENAGLR | 2 | 0.00491 | 0.00074 | 0.00451 | 0.0017875 |
| LMNA | NIYSEELR | 2 | 0.00256 | 0.0006825 | 0.00183 | 0.001425 |
| LMNA | NSNLVGAAHEELQQSR | 2 | 0.02049 | 0.0035025 | 0.01735 | 0.00429 |
| LMNA | NSNLVGAAHEELQQSR | 3 | 0.01437 | 0.000945 | 0.01343 | 0.0033575 |
| LUM | SLQDLQLTNNK | 2 | 0.01145 | 0.001285 | 0.00848 | 0.002225 |
| LUM | ILGPLSYSK | 2 | 0.0043 | 0.0004825 | 0.00272 | 0.0012725 |
| LUM | ISNIPDEYFK | 2 | 0.01165 | 0.0011925 | 0.00945 | 0.0022275 |
| LUM | FTGLQYLR | 2 | 0.0052 | 0.000725 | 0.00374 | 0.0017325 |
| MATN1 | LAVLENR | 2 | 0 | 0.0000025 | 0 | 0.0000025 |
| MATN1 | VEGLLQALTR | 2 | 0 | 0 | 0 | 0 |
| MATN1 | VGLVNYASTVKPEFPLR | 3 | 0 | 0 | 0 | 0.0001925 |
| MATN3 | SVRPLEFTK | 2 | 0 | 0.00024 | 0 | 0 |
| MATN3 | SVRPLEFTK | 3 | 0 | 0.0001925 | 0 | 0 |
| MATN3 | VAVVNYASTVK | 2 | 0 | 0.000705 | 0 | 0 |
| MATN3 | IEFQLNTYSDK | 2 | 0.00103 | 0.001945 | 0 | 0.00028 |
| MATN3 | IIDTLDIGATDTR | 2 | 0.00053 | 0.0020225 | 0 | 0.0007075 |
| MATN3 | ASGIELYAVGVDR | 2 | 0.0087 | 0.004135 | 0.00656 | 0.0018725 |
| MFGE8 | GPCSPNPCYNDAK | 2 | 0.00001 | 0.00006 | 0 | 0 |
| MFGE8 | VFQGNLDNNSHK | 2 | 0.00163 | 0.001075 | 0.00309 | 0.00109 |
| MFGE8 | VFQGNLDNNSHK | 3 | 0 | 0.0000025 | 0 | 0 |
| MFGE8 | VAYSLDGR | 2 | 0 | 0 | 0 | 0 |
| MFGE8 | QVTGIITQGAR | 2 | 0.00001 | 0.000015 | 0 | 0 |
| MFGE8 | DFGHIQYVASYK | 2 | 0.00976 | 0.0023775 | 0.01437 | 0.02439 |
| MFGE8 | DFGHIQYVASYK | 3 | 0.00241 | 0.00051 | 0.00022 | 0.000555 |
| PRELP | NLEQLR | 2 | 0 | 0 | 0 | 0 |
| PRELP | ISNVPAISNK | 2 | 0.00002 | 0.00024 | 0 | 0.0000025 |
| PRELP | LSQNLISR | 2 | 0.00034 | 0.0004475 | 0 | 0.0000125 |
| PRELP | ADTFQGLK | 2 | 0.00011 | 0.0002325 | 0 | 0.0000025 |
| PRELP | NQLEEVPSALPR | 2 | 0.00659 | 0.0010425 | 0.00416 | 0.00132 |
| DCN | NSGIENGAFQGLK | 2 | 0.02009 | 0.0022725 | 0.01327 | 0.0025425 |
| DCN | ASYSAVSLYGNPVR | 2 | 0.01781 | 0.0019675 | 0.01134 | 0.0035775 |
| DCN | ISPEAFKPLVK | 2 | 0.01208 | 0.0014825 | 0.00712 | 0.0017125 |
| DCN | ISPEAFKPLVK | 3 | 0.012 | 0.001465 | 0.00849 | 0.00187 |
| DCN | DLHTLILVNNK | 2 | 0.01218 | 0.0016575 | 0.00831 | 0.002185 |
| DCN | DLHTLILVNNK | 3 | 0.00808 | 0.001425 | 0.00488 | 0.001715 |
| CLU | VSTVTTHSSDSEVPSR | 2 | 0.01323 | 0.0021325 | 0.00434 | 0.001695 |
| CLU | VSTVTTHSSDSEVPSR | 3 | 0.01 | 0.001625 | 0.0032 | 0.0010475 |
| CLU | VTEVVVK | 2 | 0 | 0 | 0 | 0 |
| CLU | EIQNAVQGVK | 2 | 0.00576 | 0.00158 | 0.00235 | 0.00122 |
| FMOD | YLPFVPSR | 2 | 0.00001 | 0.0000525 | 0 | 0.000005 |
| FMOD | IPPVNTNLENLYLQGNR | 2 | 0.00013 | 0.00036 | 0.00012 | 0.000615 |
| FMOD | IPPVNTNLENLYLQGNR | 3 | 0.00013 | 0.00035 | 0.00002 | 0.00051 |
| OGN | ESAYLYAR | 2 | 0.00087 | 0.0005075 | 0.00001 | 0.00012 |
| OGN | LTLLNAK | 2 | 0 | 0 | 0 | 0 |
| OGN | LEGNPIALGK | 2 | 0.00001 | 0.00023 | 0 | 0.000265 |
| ASPN | TFLTTK | 2 | 0 | 0 | 0 | 0 |
| ASPN | IPSGLQELK | 2 | 0 | 0.0002175 | 0 | 0.000105 |
| ASPN | YWEIQPATFR | 2 | 0.00464 | 0.0014225 | 0.00162 | 0.00102 |
| VTN | IYVTGSLSHSAQAK | 2 | 0.00503 | 0.0009 | 0.00184 | 0.0012925 |
| VTN | IYVTGSLSHSAQAK | 3 | 0.00375 | 0.0006175 | 0.00125 | 0.000765 |
| VTN | EPQFISR | 2 | 0 | 0.0000025 | 0 | 0 |
| ACAN | VNSIYQDK | 2 | 0.00001 | 0.0002225 | 0 | 0.0000025 |
| ACAN | YPIVSPR | 2 | 0 | 0 | 0 | 0 |
| ACAN | YTLDFDR | 2 | 0 | 0 | 0 | 0 |
| ACAN | LEQFTFQEAR | 2 | 0.00131 | 0.0005725 | 0.00006 | 0.0002875 |
| COL1A1 | GFSGLDGAK | 2 | 0 | 0 | 0 | 0 |
| COL1A1 | GETGPAGPAGPIGPAGAR | 2 | 0.00045 | 0.00021 | 0.00001 | 0.0000975 |
| COL12A1 | VGVVQYSSDTR | 2 | 0 | 0.0000075 | 0 | 0.0000025 |
| COL12A1 | ALALGALQNIR | 2 | 0 | 0 | 0 | 0 |
| COL12A1 | GPGDLEAPTNLVISER | 2 | 0.00001 | 0.0001575 | 0 | 0.00041 |
| COL12A1 | IVEVFEIGPK | 2 | 0 | 0 | 0 | 0 |
| PRG4 | IVAALSIAK | 2 | 0.00322 | 0.00104 | 0.00111 | 0.00068 |
| PRG4 | DQYYNIDVPTR | 2 | 0.02456 | 0.00303 | 0.01486 | 0.00204 |
| PRG4 | GFPNVVTSAITLPNIR | 2 | 0.0335 | 0.004945 | 0.01904 | 0.00077 |
| PRG4 | GFPNVVTSAITLPNIR | 3 | 0.02319 | 0.00442 | 0.01402 | 0.00242 |
| COL9A1 | SVAFSYK | 2 | 0 | 0 | 0 | 0 |
| COL9A1 | LGSNVDFR | 2 | 0 | 0 | 0 | 0.000515 |
| COL9A1 | VVGSTALQVAYK | 2 | 0 | 0 | 0 | 0 |
| COL9A1 | GQIDADGFAVLGK | 2 | 0 | 0 | 0 | 0.00045 |
| COL11A1 | GEVGQVGPR | 2 | 0 | 0 | 0 | 0 |
| COL11A1 | LLTASAR | 2 | 0 | 0 | 0 | 0 |
| COL6A1 | VAVVQYSGQGQQQPGR | 2 | 0.00001 | 0.00004 | 0.00001 | 0.000185 |
| COL6A1 | VAVVQYSGQGQQQPGR | 3 | 0 | 0.000005 | 0 | 0.0000025 |
| COL6A1 | LKPYGALVDK | 2 | 0 | 0 | 0 | 0 |
| COL6A1 | LKPYGALVDK | 3 | 0 | 0 | 0 | 0 |
| COL6A1 | GVLYQTVSR | 2 | 0 | 0 | 0 | 0 |
| COL6A1 | LSIIATDHTYR | 2 | 0 | 0.000005 | 0 | 0.000315 |
| COL6A1 | LSIIATDHTYR | 3 | 0 | 0.0000025 | 0 | 0 |
| COL6A1 | TAEYDVAFGER | 2 | 0 | 0.000005 | 0 | 0.000005 |
| COL6A1 | VFSVAITPDHLEPR | 2 | 0 | 0.0000275 | 0 | 0.0000025 |
| COL6A1 | VFSVAITPDHLEPR | 3 | 0.00002 | 0.00011 | 0.00001 | 0.0002925 |
| COL6A1 | GLEELLIGGSHLK | 2 | 0 | 0.0000025 | 0 | 0.0000125 |
| COL6A1 | GLEELLIGGSHLK | 3 | 0 | 0.000015 | 0.00001 | 0.0000975 |
| THBS1 | LVPNPDQK | 2 | 0.00194 | 0.00103 | 0.00027 | 0.0017 |
| THBS1 | AQGYSGLSVK | 2 | 0.00148 | 0.00088 | 0 | 0.0007775 |
| THBS1 | SITLFVQEDR | 2 | 0.002 | 0.00122 | 0.00027 | 0.001175 |
| THBS1 | AGTLDLSLSLPGK | 2 | 0.00213 | 0.00095 | 0.00003 | 0.00074 |
| THBS1 | FVFGTTPEDILR | 2 | 0.00648 | 0.0014075 | 0.00325 | 0.0011525 |
| SPP1 | ISHELESSSSEVN | 2 | 0.00001 | 0.000195 | 0 | 0.0003125 |
| SPP1 | ESQESADQSDVIDSQASSK | 2 | 0.00001 | 0.0000925 | 0 | 0.0005275 |
| SPP1 | GDSLAYGLR | 2 | 0 | 0 | 0 | 0 |
| ACTBG | GYSFTTTAER | 2 |  |  |  |  |
| ACTBG | VAPEEHPVLLTEAPLNPK | 3 |  |  |  |  |
| PPIA | VSFELFADK | 2 |  |  |  |  |
| PPIA | FEDENFILK | 2 |  |  |  |  |
| TUBA | NLDIERPTYTNLNR | 3 |  |  |  |  |
| TUBA | VGINYQPPTVVPGGDLAK | 2 |  |  |  |  |
| HSP90B | FAFQAEVNR | 2 |  |  |  |  |
| HSP90B | GVVDSDDLPLNVSR | 2 |  |  |  |  |
| HSPA1A | VEIIANDQGNR | 2 |  |  |  |  |
| HSPA1A | TTPSYVAFTDTER | 2 |  |  |  |  |
| HSPA5 | ITPSYVAFTPEGER | 2 |  |  |  |  |
| HSPA5 | NELESYAYSLK | 2 |  |  |  |  |
| HSPA5 | VEIIANDQGNR | 2 |  |  |  |  |
| HSPA5 | NQLTSNPENTVFDAK | 2 |  |  |  |  |
| HSPA5 | IINEPTAAAIAYGLDK | 2 |  |  |  |  |
| HSPA5 | SQIFSTASDNQPTVTIK | 2 |  |  |  |  |
| HSPA5 | ELEEIVQPIISK | 2 |  |  |  |  |

**Table S2.** Synthesis rate of each protein-specific peptide from 25-week-old animals calculated with and without the 30-day labeling time point.

|  |  |  | **Calculated with 30-day timepoint** | | **Calculated without 30-day timepoint** | |
| --- | --- | --- | --- | --- | --- | --- |
| **Protein** | **Peptide** | **Charge** | **Synthesis rate** | **SD** | **Synthesis rate** | **SD** |
| BGN | LGLGHNQIR | 2 | 0 | 0 | 0 | 0 |
| BGN | LGLGHNQIR | 3 | 0 | 0.0000025 | 0 | 0.0000025 |
| BGN | AFSPLR | 2 | 0 | 0 | 0 | 0 |
| BGN | GVFSGLR | 2 | 0 | 0 | 0 | 0 |
| BGN | VPAGLPDLK | 2 | 0 | 0 | 0 | 0 |
| BGN | IQAIELEDLLR | 2 | 0.00254 | 0.00071 | 0.00244 | 0.0007275 |
| CHAD | EVAAGAFR | 2 | 0 | 0 | 0 | 0 |
| CHAD | LLNLQR | 2 | 0 | 0 | 0 | 0 |
| CHAD | NQLSSYPSAALSK | 2 | 0 | 0.00029 | 0 | 0.00018 |
| CHAD | SIPDNAFQSFGR | 2 | 0.00003 | 0.0002875 | 0.00003 | 0.000205 |
| CHAD | FSDAAFSGVTTLK | 2 | 0.0003 | 0.0007125 | 0.00035 | 0.00052 |
| CHAD | NNFPVLAANSFR | 2 | 0.00006 | 0.00055 | 0.00008 | 0.000425 |
| CHIL3 | TGIGAPTISTGPPGK | 2 | 0.0099 | 0.00235 | 0.0095 | 0.002455 |
| CHIL3 | DYEALNGLK | 2 | 0.00422 | 0.00285 | 0.00432 | 0.0030275 |
| CHIL3 | HLFSVLVK | 2 | 0 | 0.000495 | 0 | 0.0005575 |
| CILP | IVGPLEVNVR | 2 | 0.0021 | 0.0008025 | 0.00213 | 0.0007275 |
| CILP | FNPNAIGVPQPYLNK | 2 | 0.00978 | 0.001675 | 0.00916 | 0.0012525 |
| CILP | LVLTFVDR | 2 | 0.00501 | 0.0079675 | 0.00476 | 0.01256 |
| COL2A1 | TVIEYR | 2 | 0 | 0 | 0 | 0 |
| COL2A1 | FTYTALK | 2 | 0 | 0 | 0 | 0 |
| COL2A1 | SLNNQIESIR | 2 | 0 | 0 | 0 | 0.0000025 |
| COL2A1 | NSIAYLDEAAGNLK | 2 | 0.00001 | 0.0003 | 0.00002 | 0.00031 |
| COL6A2 | NLNEQGLR | 2 | 0 | 0 | 0 | 0 |
| COL6A2 | DIANSPHELYR | 2 | 0.00001 | 0.0001275 | 0.00001 | 0.0001825 |
| COL6A2 | DIANSPHELYR | 3 | 0 | 0.000015 | 0 | 0.0000325 |
| COL6A2 | VNSLSSFK | 2 | 0 | 0 | 0 | 0 |
| COL6A2 | LFAVAPNR | 2 | 0 | 0 | 0 | 0 |
| COL6A2 | FAYNQLIK | 2 | 0 | 0 | 0 | 0 |
| COL6A2 | VGVVQYSHEGTFEAIR | 3 | 0.00002 | 0.000155 | 0.00003 | 0.000175 |
| COL6A3 | NILTSSTGSR | 2 | 0 | 0 | 0 | 0 |
| COL6A3 | ELPNIEER | 2 | 0 | 0 | 0 | 0 |
| COL6A3 | IASNSATAFR | 2 | 0 | 0 | 0 | 0 |
| COL6A3 | DQNVFVSQK | 2 | 0 | 0 | 0 | 0 |
| COL6A3 | SSDAVAGPASSLK | 2 | 0 | 0 | 0 | 0 |
| COL6A3 | VFAVGVR | 2 | 0 | 0 | 0 | 0 |
| COL6A3 | VALVQYSDR | 2 | 0 | 0 | 0 | 0 |
| COL6A3 | VVESLDVGPDR | 2 | 0 | 0 | 0 | 0 |
| COL6A3 | ALEFVAR | 2 | 0 | 0 | 0 | 0 |
| COL6A3 | NAGPEFQYIR | 2 | 0 | 0 | 0 | 0 |
| COL6A3 | VAIAQFSDDVR | 2 | 0 | 0.000005 | 0 | 0.000005 |
| COL6A3 | ELGTIQQVISER | 2 | 0.00001 | 0.00001 | 0.00001 | 0.000015 |
| COL6A3 | VPQIAFVITGGK | 2 | 0 | 0.000015 | 0 | 0.00004 |
| COL6A3 | ISLSPEYVYSVSTFR | 2 | 0.00001 | 0.000005 | 0.00001 | 0.000005 |
| COL6A3 | IGDLQSQIVSLLK | 2 | 0.00102 | 0.000455 | 0.00097 | 0.0004825 |
| COMP | AVAEPGIQLK | 2 | 0.00462 | 0.00092 | 0.00436 | 0.000935 |
| COMP | ELQETNAALQDVR | 2 | 0.0122 | 0.00167 | 0.01149 | 0.0017675 |
| COMP | EITFLK | 2 | 0 | 0.000005 | 0.00001 | 0.0000375 |
| FN1 | GDSPASSKPVSINYK | 2 | 0.01199 | 0.00229 | 0.01142 | 0.00232 |
| FN1 | GDSPASSKPVSINYK | 3 | 0.00756 | 0.00223 | 0.00734 | 0.00213 |
| FN1 | STTPDITGYR | 2 | 0.00251 | 0.00101 | 0.00249 | 0.0010575 |
| FN1 | ITGYIIK | 2 | 0 | 0 | 0 | 0 |
| FN1 | APITGYIIR | 2 | 0.00014 | 0.000815 | 0.00034 | 0.000825 |
| FN1 | ATGVFTTLQPLR | 2 | 0.00964 | 0.0016325 | 0.00899 | 0.0015875 |
| FN1 | NLQPGSEYTVTLVAVK | 2 | 0.01707 | 0.0019525 | 0.01606 | 0.002065 |
| LMNA | ITESEEVVSR | 2 | 0.00591 | 0.00129 | 0.00543 | 0.00122 |
| LMNA | SGAQASSTPLSPTR | 2 | 0.01311 | 0.000865 | 0.01195 | 0.0008925 |
| LMNA | AAYEAELGDAR | 2 | 0.00796 | 0.001555 | 0.00748 | 0.00138 |
| LMNA | SLETENAGLR | 2 | 0.00503 | 0.0009325 | 0.00491 | 0.00074 |
| LMNA | NIYSEELR | 2 | 0.00252 | 0.0008225 | 0.00256 | 0.0006825 |
| LMNA | NSNLVGAAHEELQQSR | 2 | 0.0215 | 0.0038275 | 0.02049 | 0.0035025 |
| LMNA | NSNLVGAAHEELQQSR | 3 | 0.0157 | 0.00116 | 0.01437 | 0.000945 |
| LUM | SLQDLQLTNNK | 2 | 0.01204 | 0.00123 | 0.01145 | 0.001285 |
| LUM | ILGPLSYSK | 2 | 0.00454 | 0.0006125 | 0.0043 | 0.0004825 |
| LUM | ISNIPDEYFK | 2 | 0.01239 | 0.0012675 | 0.01165 | 0.0011925 |
| LUM | FTGLQYLR | 2 | 0.00523 | 0.000705 | 0.0052 | 0.000725 |
| MATN1 | LAVLENR | 2 | 0 | 0 | 0 | 0.0000025 |
| MATN1 | VEGLLQALTR | 2 | 0 | 0 | 0 | 0 |
| MATN1 | VGLVNYASTVKPEFPLR | 3 | 0 | 0 | 0 | 0 |
| MATN3 | SVRPLEFTK | 2 | 0 | 0.0002425 | 0 | 0.00024 |
| MATN3 | SVRPLEFTK | 3 | 0 | 0.00018 | 0 | 0.0001925 |
| MATN3 | VAVVNYASTVK | 2 | 0 | 0.0006425 | 0 | 0.000705 |
| MATN3 | IEFQLNTYSDK | 2 | 0.00082 | 0.0018275 | 0.00103 | 0.001945 |
| MATN3 | IIDTLDIGATDTR | 2 | 0.00027 | 0.0020075 | 0.00053 | 0.0020225 |
| MATN3 | ASGIELYAVGVDR | 2 | 0.00892 | 0.004585 | 0.0087 | 0.004135 |
| MFGE8 | GPCSPNPCYNDAK | 2 | 0.00001 | 0.0000575 | 0.00001 | 0.00006 |
| MFGE8 | VFQGNLDNNSHK | 2 | 0.00175 | 0.0011775 | 0.00163 | 0.001075 |
| MFGE8 | VFQGNLDNNSHK | 3 | 0 | 0.0000025 | 0 | 0.0000025 |
| MFGE8 | VAYSLDGR | 2 | 0 | 0 | 0 | 0 |
| MFGE8 | QVTGIITQGAR | 2 | 0.00001 | 0.00001 | 0.00001 | 0.000015 |
| MFGE8 | DFGHIQYVASYK | 2 | 0.01031 | 0.0024925 | 0.00976 | 0.0023775 |
| MFGE8 | DFGHIQYVASYK | 3 | 0.00244 | 0.000515 | 0.00241 | 0.00051 |
| PRELP | NLEQLR | 2 | 0 | 0 | 0 | 0 |
| PRELP | ISNVPAISNK | 2 | 0.00001 | 0.000265 | 0.00002 | 0.00024 |
| PRELP | LSQNLISR | 2 | 0.0002 | 0.0004575 | 0.00034 | 0.0004475 |
| PRELP | ADTFQGLK | 2 | 0.00002 | 0.00023 | 0.00011 | 0.0002325 |
| PRELP | NQLEEVPSALPR | 2 | 0.00694 | 0.001115 | 0.00659 | 0.0010425 |
| DCN | NSGIENGAFQGLK | 2 | 0.02204 | 0.002625 | 0.02009 | 0.0022725 |
| DCN | ASYSAVSLYGNPVR | 2 | 0.01927 | 0.002135 | 0.01781 | 0.0019675 |
| DCN | ISPEAFKPLVK | 2 | 0.01314 | 0.0017375 | 0.01208 | 0.0014825 |
| DCN | ISPEAFKPLVK | 3 | 0.01325 | 0.001635 | 0.012 | 0.001465 |
| DCN | DLHTLILVNNK | 2 | 0.01298 | 0.0015525 | 0.01218 | 0.0016575 |
| DCN | DLHTLILVNNK | 3 | 0.00887 | 0.00136 | 0.00808 | 0.001425 |
| CLU | VSTVTTHSSDSEVPSR | 2 | 0.01395 | 0.00255 | 0.01323 | 0.0021325 |
| CLU | VSTVTTHSSDSEVPSR | 3 | 0.01052 | 0.0016025 | 0.01 | 0.001625 |
| CLU | VTEVVVK | 2 | 0 | 0 | 0 | 0 |
| CLU | EIQNAVQGVK | 2 | 0.00613 | 0.0016725 | 0.00576 | 0.00158 |
| FMOD | YLPFVPSR | 2 | 0.00001 | 0.00008 | 0.00001 | 0.0000525 |
| FMOD | IPPVNTNLENLYLQGNR | 2 | 0.0001 | 0.000355 | 0.00013 | 0.00036 |
| FMOD | IPPVNTNLENLYLQGNR | 3 | 0.00011 | 0.0003625 | 0.00013 | 0.00035 |
| OGN | ESAYLYAR | 2 | 0.00054 | 0.0004475 | 0.00087 | 0.0005075 |
| OGN | LTLLNAK | 2 | 0 | 0 | 0 | 0 |
| OGN | LEGNPIALGK | 2 | 0.00001 | 0.0002275 | 0.00001 | 0.00023 |
| ASPN | TFLTTK | 2 | 0 | 0 | 0 | 0 |
| ASPN | IPSGLQELK | 2 | 0 | 0.0001325 | 0 | 0.0002175 |
| ASPN | YWEIQPATFR | 2 | 0.00453 | 0.001695 | 0.00464 | 0.0014225 |
| VTN | IYVTGSLSHSAQAK | 2 | 0.00501 | 0.00093 | 0.00503 | 0.0009 |
| VTN | IYVTGSLSHSAQAK | 3 | 0.00365 | 0.0007725 | 0.00375 | 0.0006175 |
| VTN | EPQFISR | 2 | 0 | 0.0000025 | 0 | 0.0000025 |
| ACAN | VNSIYQDK | 2 | 0.00001 | 0.00018 | 0.00001 | 0.0002225 |
| ACAN | YPIVSPR | 2 | 0 | 0 | 0 | 0 |
| ACAN | YTLDFDR | 2 | 0 | 0 | 0 | 0 |
| ACAN | LEQFTFQEAR | 2 | 0.00121 | 0.000675 | 0.00131 | 0.0005725 |
| COL1A1 | GFSGLDGAK | 2 | 0 | 0 | 0 | 0 |
| COL1A1 | GETGPAGPAGPIGPAGAR | 2 | 0.0004 | 0.0002175 | 0.00045 | 0.00021 |
| COL12A1 | VGVVQYSSDTR | 2 | 0 | 0.0000025 | 0 | 0.0000075 |
| COL12A1 | ALALGALQNIR | 2 | 0 | 0.0000025 | 0 | 0 |
| COL12A1 | GPGDLEAPTNLVISER | 2 | 0.00001 | 0.00018 | 0.00001 | 0.0001575 |
| COL12A1 | IVEVFEIGPK | 2 | 0 | 0 | 0 | 0 |
| PRG4 | IVAALSIAK | 2 | 0.00337 | 0.0012475 | 0.00322 | 0.00104 |
| PRG4 | DQYYNIDVPTR | 2 | 0.02677 | 0.0029675 | 0.02456 | 0.00303 |
| PRG4 | GFPNVVTSAITLPNIR | 2 | 0.03449 | 0.0033875 | 0.0335 | 0.004945 |
| PRG4 | GFPNVVTSAITLPNIR | 3 | 0.02483 | 0.00397 | 0.02319 | 0.00442 |
| COL9A1 | SVAFSYK | 2 | 0 | 0 | 0 | 0 |
| COL9A1 | LGSNVDFR | 2 | 0 | 0 | 0 | 0 |
| COL9A1 | VVGSTALQVAYK | 2 | 0 | 0 | 0 | 0 |
| COL9A1 | GQIDADGFAVLGK | 2 | 0 | 0 | 0 | 0 |
| COL11A1 | GEVGQVGPR | 2 | 0 | 0 | 0 | 0 |
| COL11A1 | LLTASAR | 2 | 0 | 0 | 0 | 0 |
| COL6A1 | VAVVQYSGQGQQQPGR | 2 | 0.00001 | 0.000025 | 0.00001 | 0.00004 |
| COL6A1 | VAVVQYSGQGQQQPGR | 3 | 0 | 0.000005 | 0 | 0.000005 |
| COL6A1 | LKPYGALVDK | 2 | 0 | 0 | 0 | 0 |
| COL6A1 | LKPYGALVDK | 3 | 0 | 0 | 0 | 0 |
| COL6A1 | GVLYQTVSR | 2 | 0 | 0 | 0 | 0 |
| COL6A1 | LSIIATDHTYR | 2 | 0 | 0.0000025 | 0 | 0.000005 |
| COL6A1 | LSIIATDHTYR | 3 | 0 | 0.0000025 | 0 | 0.0000025 |
| COL6A1 | TAEYDVAFGER | 2 | 0 | 0.0000025 | 0 | 0.000005 |
| COL6A1 | VFSVAITPDHLEPR | 2 | 0 | 0.0000075 | 0 | 0.0000275 |
| COL6A1 | VFSVAITPDHLEPR | 3 | 0.00001 | 0.000125 | 0.00002 | 0.00011 |
| COL6A1 | GLEELLIGGSHLK | 2 | 0 | 0.0000025 | 0 | 0.0000025 |
| COL6A1 | GLEELLIGGSHLK | 3 | 0 | 0.0000075 | 0 | 0.000015 |
| THBS1 | LVPNPDQK | 2 | 0.00192 | 0.0012375 | 0.00194 | 0.00103 |
| THBS1 | AQGYSGLSVK | 2 | 0.00128 | 0.00092 | 0.00148 | 0.00088 |
| THBS1 | SITLFVQEDR | 2 | 0.00204 | 0.001375 | 0.002 | 0.00122 |
| THBS1 | AGTLDLSLSLPGK | 2 | 0.00222 | 0.001165 | 0.00213 | 0.00095 |
| THBS1 | FVFGTTPEDILR | 2 | 0.00695 | 0.00175 | 0.00648 | 0.0014075 |
| SPP1 | ISHELESSSSEVN | 2 | 0.00001 | 0.0001475 | 0.00001 | 0.000195 |
| SPP1 | ESQESADQSDVIDSQASSK | 2 | 0.00001 | 0.0001325 | 0.00001 | 0.0000925 |
| SPP1 | GDSLAYGLR | 2 | 0 | 0 | 0 | 0 |
